# Comparative Small RNA and Degradome Sequencing Provides Insights into Antagonistic Interactions in the Biocontrol Fungus Clonostachys rosea

**DOI:** 10.1128/aem.00643-22

**Published:** 2022-06-13

**Authors:** Edoardo Piombo, Ramesh Raju Vetukuri, Poorva Sundararajan, Sandeep Kushwaha, Dan Funck Jensen, Magnus Karlsson, Mukesh Dubey

**Affiliations:** a Department of Forest Mycology and Plant Pathology, Swedish University of Agricultural Sciences, Uppsala, Sweden; b Department of Plant Breeding, Horticum, Swedish University of Agricultural Sciences, Lomma, Sweden; c National Institute of Animal Biotechnology, Hyderabad, Telangana, India; Nanjing Agricultural University

**Keywords:** antagonism, biocontrol, *Clonostachys rosea*, degradome, fungus-fungus interaction, gene regulation, mycoparasitism, posttranscriptional gene silencing, RNA interference, small-RNAs

## Abstract

Necrotrophic mycoparasitism is an intricate process involving recognition, physical mycelial contact, and killing of host fungi (mycohosts). During such interactions, mycoparasites undergo a complex developmental process involving massive regulatory changes of gene expression to produce a range of chemical compounds and proteins that contribute to the parasitism of the mycohosts. Small RNAs (sRNAs) are vital components of posttranscriptional gene regulation, although their role in gene expression regulation during mycoparasitisms remain understudied. Here, we investigated the role of sRNA-mediated gene regulation in mycoparasitism by performing sRNA and degradome tag sequencing of the mycoparasitic fungus Clonostachys rosea interacting with the plant-pathogenic mycohosts Botrytis cinerea and Fusarium graminearum at two time points. The majority of differentially expressed sRNAs were downregulated during the interactions with the mycohosts compared to a *C. rosea* self-interaction control, thus allowing desuppression (upregulation) of mycohost-responsive genes. Degradome analysis showed a positive correlation between high degradome counts and antisense sRNA mapping and led to the identification of 201 sRNA-mediated potential gene targets for 282 differentially expressed sRNAs. Analysis of sRNA potential gene targets revealed that the regulation of genes coding for membrane proteins was a common response against both mycohosts. The regulation of genes involved in oxidative stress tolerance and cellular metabolic and biosynthetic processes was exclusive against F. graminearum, highlighting common and mycohost-specific gene regulation of *C*. *rosea*. By combining these results with transcriptome data collected during a previous study, we expand the understanding of the role of sRNA in regulating interspecific fungal interactions and mycoparasitism.

**IMPORTANCE** Small RNAs (sRNAs) are emerging as key players in pathogenic and mutualistic fungus-plant interactions; however, their role in fungus-fungus interactions remains elusive. In this study, we employed the necrotrophic mycoparasite *Clonostachys rosea* and the plant-pathogenic mycohosts Botrytis cinerea and Fusarium graminearum and investigated the sRNA-mediated gene regulation in mycoparasitic interactions. The combined approach of sRNA and degradome tag sequencing identified 201 sRNA-mediated putative gene targets for 282 differentially expressed sRNAs, highlighting the role of sRNA-mediated regulation of mycoparasitism in *C. rosea.* We also identified 36 known and 13 novel microRNAs (miRNAs) and their potential gene targets at the endogenous level and at a cross-species level in *B. cinerea* and F. graminearum, indicating a role of cross-species RNA interference (RNAi) in mycoparasitism, representing a novel mechanism in biocontrol interactions. Furthermore, we showed that *C*. *rosea* adapts its transcriptional response, and thereby its interaction mechanisms, based on the interaction stages and identity of the mycohost.

## INTRODUCTION

RNA interference (RNAi) is a method of gene expression regulation based on small RNAs (sRNAs), which can influence gene regulation at the transcriptional and posttranscriptional level (1, 2). These sRNAs usually have a length of 18 to 40 nucleotides, and their silencing action is mediated mainly by three categories of enzymes: dicer or dicer-like endoribonucleases (DCLs), Argonaute (AGO) proteins, and RNA-dependent RNA polymerases (RDRPs). The role of DCLs is to cleave double-stranded RNA precursors, generating several categories of sRNAs, the most studied of which is microRNAs (miRNAs, milRNAs in fungi). These are small noncoding RNAs of 18 to 26 nucleotides, normally generated from single-stranded RNA forming hairpin structures (3).

These units are then recognized by the RNA-induced silencing complex (RISC) and used as a guide by AGO proteins to cleave or inhibit the translation of transcripts showing complementarity to the sRNAs (4). As a final step, RDRPs generate additional double-stranded RNAs from sRNAs, amplifying the silencing signal (5, 6). PhasiRNAs, extensively found in plants, are sRNAs of 21 to 26 nucleotides in size generated from the Dicer-driven cleavage of long precursors synthesized by RDRP enzymes, acting on the cleaved targets of specific miRNAs (7, 8). sRNA-mediated transcript cleavage results in a rapidly degraded upstream fragment and a stable downstream fragment (9), and the resulting products of sRNA-directed transcript cleavage can be determined through the sequencing of the 5′ ends of uncapped polyadenylated mRNAs. Parallel analysis of RNA ends (PARE) (10), also known as degradome sequencing, is a well-known technique to identify sRNA gene targets and cleavage sites by mapping the degraded reads on mRNA transcripts, and nucleotide base complementarity between the transcript and sRNA at the 10th and 11th positions is used to identify degradation peaks (11). Degradome sequencing combined with bioinformatic analysis has been used to identify candidate sRNAs and their putative gene target in plants (10, 12, 13) as well as in fungi such as Fusarium graminearum and Rhizophagus irregularis (14, 15).

RNA interference has been observed to influence multiple processes at the endogenous level in fungi, such as sexual reproduction in F. graminearum (14), response to cellulose in Trichoderma reesei (16), and mycelial growth and conidiogenesis in Metarhizium anisopliae (17) and Trichoderma atroviride (18). In addition to their endogenous role, specific sRNAs can travel between interacting species and regulate the function of recipient cells by hijacking their RNAi machinery (19, 20). Furthermore, RNAi has recently been exploited to control plant pathogens by exogenous application of sRNAs targeting genes in pathogens essential for disease development by a process called spray-induced gene silencing (21–23).

Clonostachys rosea is an ascomycete fungus known for its mycoparasitic and antagonistic ability against several plant-pathogenic fungi (24) and nematodes (25). Therefore, certain strains of *C. rosea* are commercialized and used for the biological control of plant diseases in crop production (24). The antagonistic ability of *C. rosea* is achieved through the production of specialized metabolites (26–29), hydrolytic enzymes (30–33), and other secreted proteins (34, 35). In addition, *C. rosea* possess numerous drug resistance membrane transporters that can mediate the expulsion of both endogenous and exogenous toxic compounds (36–40). During antagonistic interactions with other fungi, *C*. *rosea* can recognize its mycohosts and respond with both common and specific transcriptional changes (39), demonstrating a mycohost-dependent expression of the genetic machinery. However, the issue of how these changes in gene expression are mediated remained elusive until recent work demonstrated a role of DCL-mediated RNAi in antagonistic interactions in *C. rosea* (41).

The aim of this study was to (i) expand the understanding of RNAi-mediated antagonistic interactions in *C. rosea* by identifying candidate sRNAs and their cleavage products (gene targets) at endogenous (within *C. rosea*) and cross-species (in mycohosts) levels and (ii) to investigate if, and to what extent, *C*. *rosea* deploys common or mycohost-specific sRNAs in regulating antagonistic interactions. To achieve these objectives, we sequenced both sRNAs and degradomes of *C. rosea* during two stages of interaction with two intrinsically and phylogenetically different plant-pathogenic fungi (mycohosts), Botrytis cinerea and F. graminearum. By combining the results from sRNA and degradome sequencing with transcriptome data collected in similar experimental conditions during a previous study, we identified candidate sRNAs (including known and novel milRNAs) and their putative gene targets potentially associated with antagonistic interactions in *C. rosea*. This includes the identification of pathogen genes putatively cleaved by *C. rosea* sRNAs, already predicted in a previous study (41), indicative of cross-species transfer of sRNAs. Furthermore, comparative/combined sRNA and degradome analyses revealed that *C. rosea* can modulate its regulatory network depending on mycohosts and stages of non-self-interactions.

## RESULTS

### Antagonistic effect of *C. rosea* against *B. cinerea* and F. graminearum.

**FIG 1:**
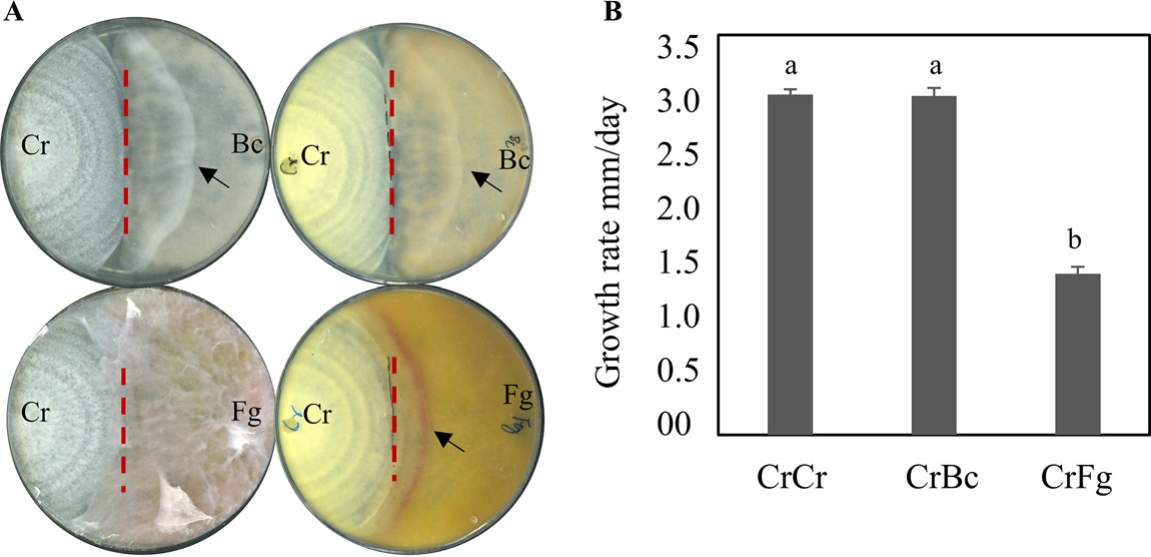
Measuring the antagonistic ability of *C. rosea* against *B. cinerea* and F. graminearum using an *in vitro* dual culture plate confrontation assay. (A) An agar plug of *C*. *rosea* (Cr, left side of the plate) strains against *B. cinerea* (Bc) or F. graminearum (Fg, right side of the plate) was inoculated on opposite sides in 9-cm-diameter agar plates and incubated at 25°C. The experiment was performed in four replicates, and photographs of representative plates were taken. An arrowhead indicates the *C. rosea* mycelial front; the dashed line indicates the point of mycelial contact. (B) Growth rate (overgrow) of *C*. *rosea* on *B*. *cinerea* and F. graminearum. The growth of *C*. *rosea* on mycohosts was measured from the point of mycelial contact. Error bars represent the standard deviation based on four biological replicates. Different letters indicate statistically significant differences based on Tukey’s honestly significant difference (HSD) method at the 95% significance level. Cr-Cr, self-interaction control; CrBc, interaction with Botrytis cinerea, CrFg, interaction with F. graminearum.

The antagonistic ability of *C*. *rosea* toward *B*. *cinerea* and F. graminearum was assessed by measuring the mycelial growth rate of interacting species in an *in vitro* dual culture-plate confrontation assay (see Fig. S1 in the supplemental material). The growth rates of each fungus grown alone (noninteraction) and against itself (self-interaction) were used as controls. In comparison to the noninteraction control, no significant changes in mycelial growth rates of *C. rosea* or *B*. *cinerea* were found during self-interactions or non-self-interactions (Fig. S2). In contrast, F. graminearum showed a significant (*P ≤ *0.017) reduction in growth rate during non-self- (CrFg) and self-interactions (FgFg) compared to the noninteraction (Fg) control 3 days postinoculation (dpi). After 4 days of incubation, the mycelial growth rate of F. graminearum in non-self-interaction was reduced by 23% (*P = *0.010) compared to the noninteraction control (Fig. S2). After 4 dpi, the mycelial fronts of F. graminearum during self-interaction merged, thereby preventing further measurements. The result is in line with the previous finding of Zapparata et al. (42), where a lower growth rate of F. graminearum is also reported during self-interaction than when grown alone. After mycelial contact, *C. rosea* overgrew the mycelium of *B*. *cinerea* with the same rate as the *C. rosea* noninteracting control. In contrast, there was a significant (*P = *0.001) 53% reduction in the *C. rosea* overgrowth rate on F. graminearum mycelium compared to the growth rate in the noninteraction control or overgrowth on *B. cinerea* (Fig. 1).

### Deep sequencing of *C. rosea* sRNAs.

A dual culture-plate confrontation assay was used for total RNA extraction of *C*. *rosea* during *in vitro* interaction with two mycohosts, *B*. *cinerea* (CrBc) and F. graminearum (CrFg). Mycelial fronts were harvested at two stages, at mycelial contact and 24 h after mycelial contact. *C*. *rosea* interacting with itself (CrCr) was used as a control treatment (Fig. S1). sRNA sequencing generated a total of 1,052 million read pairs, ranging between 156 million to 192 million read pairs per treatment depending on the treatments (Table S1). After trimming of adaptor sequences, 969 million reads were obtained. The reads originating from *B*. *cinerea* and F. graminearum were removed by mapping sRNA reads to the *C*. *rosea*, *B*. *cinerea*, and F. graminearum genomes, allowing zero mismatch. A total of 192 million read pairs from CrBc and CrFg unique to *C*. *rosea* (mapping to the *C*. *rosea* genome and not to *B*. *cinerea* or F. graminearum) remained. Based on the sRNA length that was previously observed for sRNAs in fungi (43), reads of 18 to 32 nucleotides long were used for further analyses.

### Origin and characteristics of *C. rosea* sRNAs.

**FIG 2:**
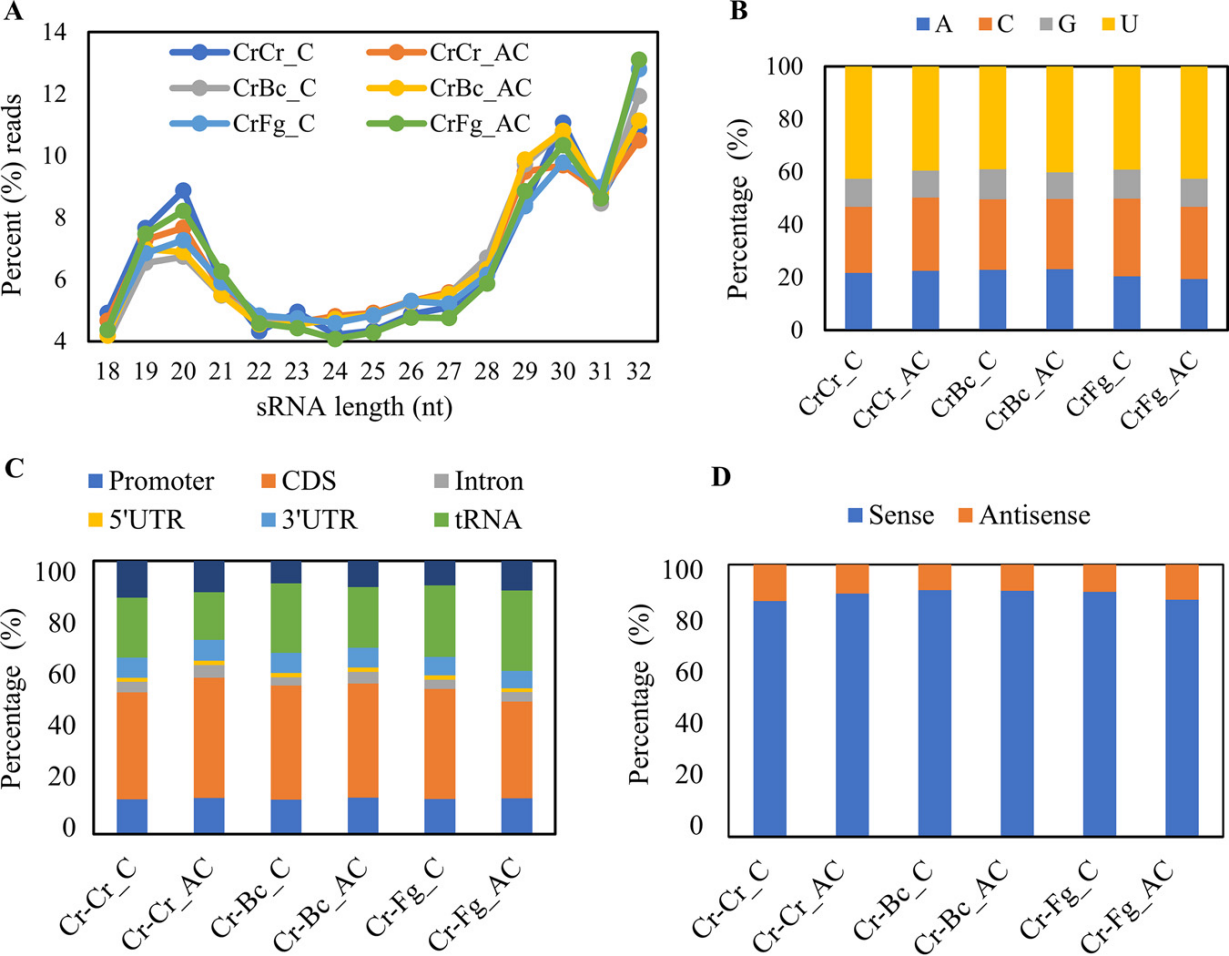
Characteristics of sRNAs in *C. rosea* interacting with *B. cinerea* (CrBc) and F. graminearum (CrFg) and in the controls (CrCr), both at contact (C) and after contact (AC) stages of interaction. (A) Length distribution; (B) 5′ nucleotide percentages; (C) location mapping; (D) sense mapping.

Analysis of sRNA length distribution showed no apparent differences in the proportion of size distribution of reads between the treatments. The 32-nucleotide (nt) and 18-nt lengths represented the highest and lowest proportion of reads, respectively, in all the treatments (Fig. 2A). The analysis of the 5′-terminal nucleotide composition showed that a higher proportion of sRNA reads (42.6%) starts with uracil (5′-U) during the contact stage of CrCr self-interaction compared with CrBc (39.0%) and CrFg (39.1%) (Fig. 2B). To investigate the origin of sRNAs, we mapped sRNA sequences to the *C*. *rosea* genome. Our result showed that 70 to 76% of sRNA sequences originated from exons (coding sequences [CDSs], 5′ untranslated regions [5′ UTRs], and 3′ UTRs), followed by tRNAs, promoters, and intergenic regions (Fig. 2C). A lower proportion (35%) of sRNA reads was found to originate from CDSs 24 h after contact with F. graminearum (CrFg), compared with 44% during CrCr self-interaction at the same stage. In addition, we analyzed the relative proportion of sRNAs putatively originating from exons for their mapping to the sense or antisense strand of exons. Out of the total RNAs mapping on exons, 89% and 11% of sRNA reads were mapped to the sense and antisense strand, respectively (Fig. 2D). These results are in line with the previous findings obtained for Mucor circinelloides (44), *F. graminearum* (14), *T. atroviride* (18) and the arbuscular mycorrhizal fungus *Rhizophagus irregularis* (15), where sense strands of exons are shown to be the major source of sRNA origins.

### sRNA expression in *C. rosea* is both mycohost and interaction stage dependent.

To identify sRNAs differentially expressed in *C*. *rosea* during non-self-interactions, antisense-specific sRNAs and sRNAs potentially originating from intergenic and intronic regions were selected for differential expression analysis. Antisense-specific sRNAs were selected due to the reported high correlation between a high mapping of antisense sRNAs and high transcript degradation (14). On the other hand, sRNAs originated from intergenic and intronic regions were selected due to previous findings where a majority of predicted *C. rosea* milRNAs were shown to locate in intergenic regions (41), and intron-containing mRNA precursors were shown to template siRNA synthesis in Cryptococcus neoformans (45). A summary of differentially expressed sRNAs is provided in Data Set S1.

**FIG 3:**
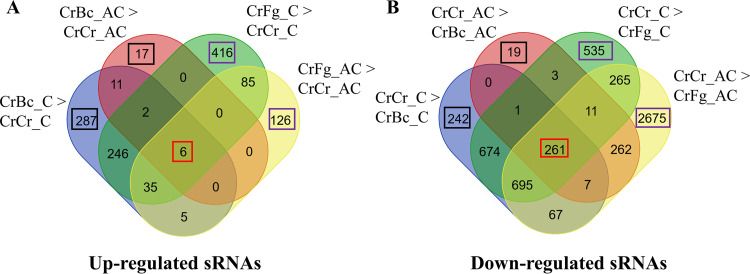
Venn diagram showing common and mycohost-specific expression of sRNAs during *C. rosea* interactions with *B. cinerea* (CrBc) and F. graminearum (CrFg) compared to the self interaction control (CrCr) at contact (C) and after contact (AC) stages of interactions. (A) Upregulated sRNAs; (B) downregulated sRNAs. sRNAs differentially expressed in every situation are outlined in red, while sRNAs differentially expressed only in response to one condition are outlined in black (*B. cinerea*) or purple (F. graminearum).

The expression profile of sRNAs during CrBc and CrFg was compared to that of the CrCr control at the respective time points. In comparison to the CrCr control, 1,947 and 564 sRNAs were downregulated at contact and after contact stages of CrBc, respectively, while a lower number of sRNAs (590 and 36) were upregulated under the same conditions. Among these, 269 downregulated and 19 upregulated sRNAs were common between both interaction stages (Fig. 3). During the CrFg interactions, 2,445 and 4,250 sRNAs were downregulated at contact and after contact stages, compared with CrCr control, while 790 and 257 were upregulated under the same conditions (Fig. 3). In summary, our data showed that differential sRNA expression in *C. rosea* is partially dependent on the stage of the interaction, and the number of downregulated sRNAs was higher than the number of upregulated sRNAs.

Transcriptome (mRNA) analysis in *C*. *rosea* during CrBc and CrFg interactions showed common and species-specific responses toward the mycohosts (39). We investigated whether this finding held for sRNA expression and found that a higher number of downregulated sRNAs (1,631) was common at the contact stage against both mycohosts, while 316 and 814 sRNAs were specifically downregulated against *B. cinerea* or F. graminearum, respectively.

After contact, 541 sRNAs were commonly downregulated against both the mycohosts, while 23 and 3,702 were downregulated only against *B. cinerea* or F. graminearum, respectively. Among the upregulated sRNAs, 289 and 6 sRNAs were commonly upregulated against both the mycohosts at contact and after contact, respectively (Fig. 3). In summary, sRNA expression analysis showed that more *C. rosea* sRNAs were differentially expressed in CrFg than during CrBc. In addition, the number of differentially expressed sRNAs is higher at the contact stage in CrBc, but at the after contact stage in CrFg. Therefore, differential sRNA expression in *C. rosea* depends on both mycohost identity and interaction stage (Fig. 3).

**FIG 4:**
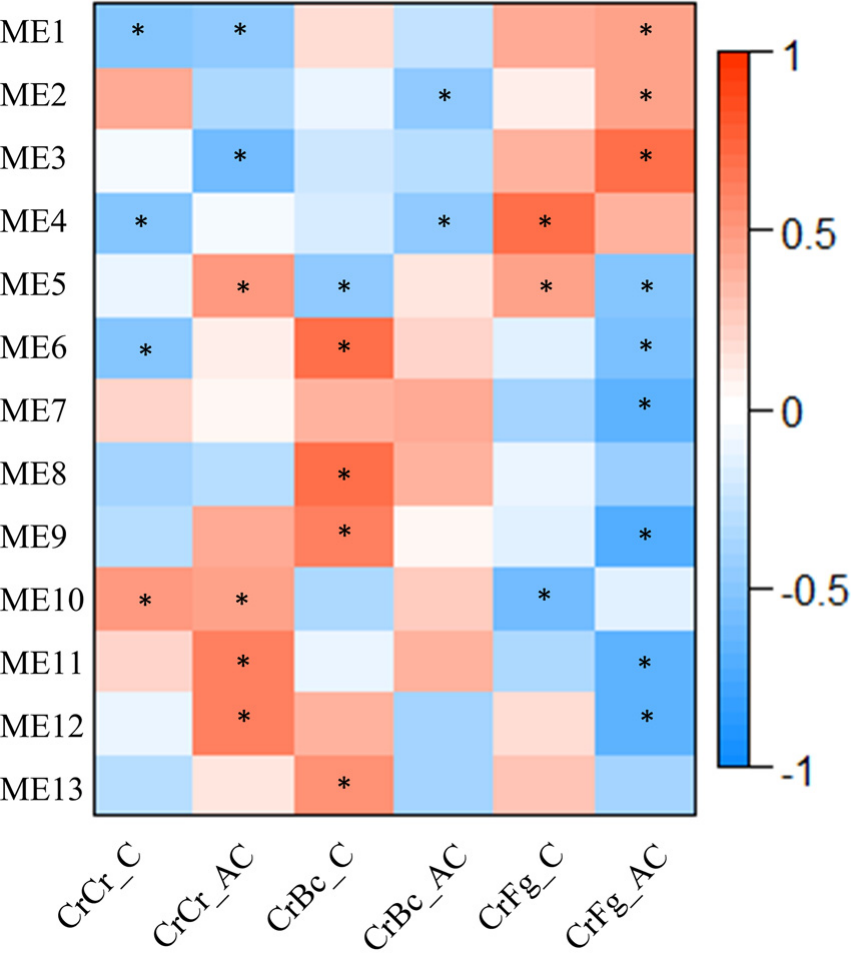
The heatmap shows the Spearman correlation between the module eigengenes of coexpression modules generated with WGCNA and the conditions examined in this study. *C. rosea* interaction with *B. cinerea* (CrBc), F. graminearum (CrFg), and the controls (CrCr), both at contact (C) and after contact (AC) stages of interactions. Asterisks indicate significant correlations between the coexpression modules and the conditions examined in this study. The modules were generated using the normalized expression values of differentially expressed sRNAs.

This result was further validated by a coexpression analysis executed with weighted correlation network analysis (WGCNA), which divided the differentially expressed sRNAs into 13 modules. The module eigengenes (ME), which are the first principal components of the expression matrix of each module, were shown to correlate to *B. cinerea* in the case of ME 6, 7, and 8 and to F. graminearum in the case of ME 3 and 4. ME 12 and 13 responded to the interaction stage (Fig. 4).

Differentially expressed sRNAs were mapped to the *C. rosea* genome to investigate whether these sRNAs originated from a specific region in the genome. sRNAs upregulated at the contact stage of both mycohosts and after contact with F. graminearum mainly originated from a precise group of scaffolds (Fig. S3). The sRNAs upregulated after contact with *B. cinerea* were too few to analyze. The downregulated sRNAs had an even more specific origin, in most cases originating from either scaffold unitig_227 or unitig_242 as can be seen from the heatmap in Fig. S3.

### Identification and expression analysis of *C. rosea* milRNAs.

We used MiRDeep2 (46) for milRNA prediction and identified 36 known and 13 novel milRNAs (Data Set 2). A summary of milRNA sequences, origins, precursors, and abundance is provided in Data Set 2. All 49 milRNAs had their reverse complement detected among the clean reads. In CrBc, five milRNAs (cro-mir-1, cro-mir-36, cro-mir-62, cro-mir-63, and cro-mir-70) were downregulated, while two milRNAs (cro-mir-4 and cro-mir-72) were upregulated (Table 1). In CrFg, three milRNAs (cro-mir-1, cro-mir-36, and cro-mir-63) were downregulated and five (cro-mir-8, cro-mir-9, cro-mir-23, cro-mir-34, and cro-mir-72) were upregulated. Three (cro-mir-1, cro-mir-36, and cro-mir-63) of the downregulated milRNAs were common to both CrBc and CrFg, while cro-mir-72 was upregulated commonly to both interactions (Table 1). The expression of a subset of sRNAs and miRNAs was further confirmed through stem-loop reverse transcription quantitative PCR (RT-qPCR; Table 2). In summary, the number of differentially expressed milRNAs at the contact stage of interaction was higher than the number of differentially expressed milRNAs after the contact stage (6 milRNAs; Table 1). Among the differentially expressed milRNAs, four (cro-mir-1, cro-mir-4, cro-mir-9, and cro-mir-36) were proven to be DCL2-dependent as reported in a previous study of Piombo et al. (41). Their expression and predicted gene targets (41) are presented in Table 3.

**TABLE 1:** Novel and known milRNAs detected in *C. rosea* and their expression patterns during non-self-interactions with *B. cinerea* or *F. graminearium* compared to the self-interaction control^*a*^

milRNA	Mature sequence	Length (nt)	Log_2_ FC expression^*c*^			
			CrBc_C	CrBc_AC	CrFg_C	CrFg_AC
cro-mir-62	gaucgcuaucacuuggau	18	0.48	**–1.48**	0.41	–0.52
cro-mir-63	uguuaugaugcacaguaccugaga	24	**–1.65**	–0.4	**–1.84**	**–1.75**
cro-mir-70	uugagcgcgcucuugcugcu	20	**–2.79**	–0.77	−1.02	0.79
cro-mir-72	uugguuagcguacgagacu	19	**3.10**	0.54	**2.77**	1.49
cro-mir-1^*b*^	uagaauucgggguagaau	18	**–2.09**	**–1.15**	**–1.77**	**–1.76**
cro-mir-4^*b*^	ucagccucgagacuuugcc	19	**1.13**	0.31	0.81	–0.08
cro-mir-8^*b*^	acccugucguugucgcca	18	–0.45	0.23	0.6	**3.19**
cro-mir-9^*b*^	ucggacguauauugacuacuc	21	1.19	0.21	**1.47**	1.27
cro-mir-23^*b*^	ctggcaggtatggtcgtagatg	22	0.86	0.76	0.6	**1.33**
cro-mir-34^*b*^	uuggagucagacaugaaguc	20	2.67	2.06	**4.06**	3
cro-mir-36^*b*^	ucaaacacaauuagcgguc	19	**–1.75**	–0.8	**–1.16**	–0.39

a CrBc, *C. rosea* interaction with Botrytis cinerea; CrFg, *C. rosea* interaction with Fusarium graminearum; C, contact stage; AC, after contact stage.

b Known milRNA reported by Piombo et al. (41).

c Significant differences (FDR = 0.05 and log2 fold change >1) are highlighted in boldface.

**TABLE 2:** Validation of sRNA sequencing through stem-loop RT-qPCR^*a*^

sRNA ID	Primer sequences	Log_2_ FC sequencing		Log_2_ FC stem-loop RT-qPCR	
		CrBc_C	CrFg_C	CrBc_C	Cr-Fg_C
ae_seq_277875_x17263	SLP: gttggctctggtgcagggtccgaggtattcgcaccagagccaacccttcc	–4.06	–3.4	–4.02	–3.92
	FW: cggcggtggtcgagctga				
ii_seq_8561252_x801	SLP: gttggctctggtgcagggtccgaggtattcgcaccagagccaactaaata	–3.96	–4.03	–2.82	–5.29
	FW: cggcggatcgataagctgtg				
cro-mir-1	SLP: gttggctctggtgcagggtccgaggtattcgcaccagagccaacattcta	–2.08	–1.77	–3.98	–2.27
	FW: cggcggtagaattcgggg				
cro-mir-36	SLP: gttggctctggtgcagggtccgaggtattcgcaccagagccaacgaccgc	–1.75	–1.16	–1.82	–3.04
	FW: cggcggtcaaacacaatta				
cro-mir-72	SLP: gttggctctggtgcagggtccgaggtattcgcaccagagccaacagtctc	3.1	2.77	0.56	0.48
	FW: cggcggttggttagcgtac				
Universal reverse primer	gtgcagggtccgaggt				

a CrBc, *C. rosea* interaction with Botrytis cinerea; CrFg, *C. rosea* interaction with Fusarium graminearum; C, contact stage; AC, after contact stage; SLP, stem-loop RT-qPCR primer; FW, forward RT-qPCR primer. A subset of sRNAs and miRNAs were selected for the validation.

**TABLE 3:** Differential expression of DCL2-dependent milRNAs and their gene targets^*a*^

milRNA^*c*^	Log_2_ FC sRNA expression^*b*^				Putative gene targets	Annotation
	CrBc_C	CrBc_AC	CrFg_C	CrFg_AC		
Endogenous gene targets						
cro-mir-1	**–2.08**	**–1.14**	**–1.77**	**–1.76**	CRV2T00003756	Aminoacyl-tRNA ligase
cro-mir-1	**–2.08**	**–1.14**	**–1.77**	**–1.76**	CRV2T00017618	Uncharacterized
cro-mir-36	**–1.75**	–0.8	**–1.16**	–0.39	CRV2T00011823	Sulfuric ester hydrolase
cro-mir-36	**–1.75**	–0.8	**–1.16**	–0.39	CRV2T00013380	ATPase
cro-mir-36	**–1.75**	–0.8	**–1.16**	–0.39	CRV2T00005499	Uncharacterized
cro-mir-36	**–1.75**	–0.8	**–1.16**	–0.39	CRV2T00000111	Uncharacterized
cro-mir-36	**–1.75**	–0.8	**–1.16**	–0.39	CRV2T00014914	Uncharacterized
cro-mir-36	**–1.75**	–0.8	**–1.16**	–0.39	CRV2T00000903	Uncharacterized
cro-mir-36	**–1.75**	–0.8	**–1.16**	–0.39	CRV2T00004261	Uncharacterized
cro-mir-4	**1.13**	0.31	0.81	–0.08	CRV2T00011242	Uncharacterized
cro-mir-9	1.19	0.21	**1.47**	1.27	CRV2T00004339	SNF2 family helicase
Cross-species gene targets in *B. cinerea*						
cro-mir-4	**1.13**	0.31	0.81	–0.08	XM_024690414.1	Chitin synthase BcCHSIV
cro-mir-4	**1.13**	0.31	0.81	–0.08	XM_024693385.1	Serine threonine-protein phosphatase
cro-mir-4	**1.13**	0.31	0.81	–0.08	XM_024696641.1	Vacuolar sorting protein Bcvps27
cro-mir-4	**1.13**	0.31	0.81	–0.08	XM_024695521.1	Chromatin binding factor Bcyta7
cro-mir-4	**1.13**	0.31	0.81	–0.08	XM_001556587.2	Transcription factors bcltf3
Cross-species gene targets in F. graminearum						
cro-mir-9	1.19	0.21	**1.47**	1.27	XM_011320613.1	Zinc-binding protein
cro-mir-9	1.19	0.21	**1.47**	1.27	XM_011329717.1	Sec1 family vesicle transport protein

a Cr, *C*. *rosea*; Bc, *B*. *cinerea*; Fg, F. graminearum; C, contact stage; AC, after contact stage.

b Significant differences (FDR = 0.05 and log_2_ fold change > 1) are highlighted in boldface.

c These milRNAs were significantly downregulated in *C. rosea* Δ*dcl2* strains, and consequently, the expression of their gene targets was upregulated as reported by Piombo et al. (41).

### Degradome analysis showed a positive correlation between high degradome counts and antisense sRNA mapping.

**FIG 5:**
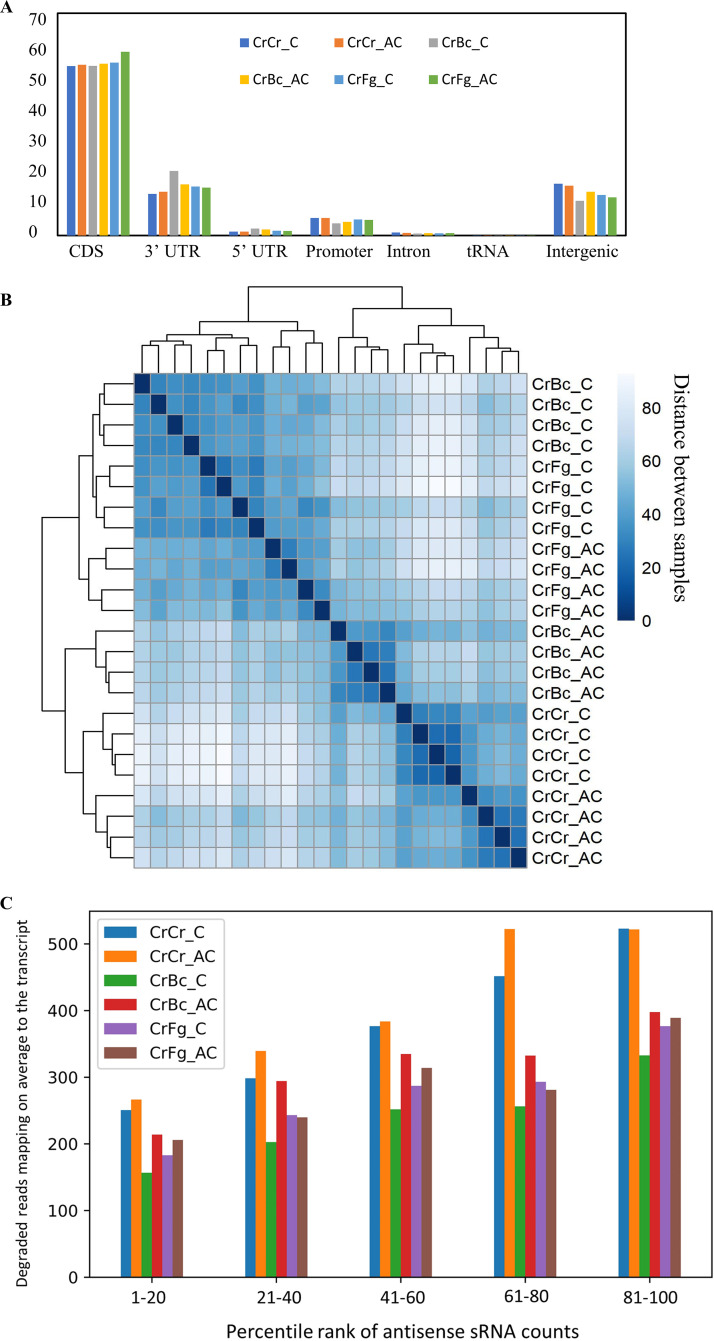
Degradome sequence analyses. (A) Results of degradome read mapping on gene features. The reads belong to *C. rosea* interacting with *B. cinerea* (CrBc), F. graminearum (CrFg), and controls (CrCr). (B) Hierarchical clustering of samples depending on degradome read mapping to *C. rosea* transcripts. (C) Average degraded read count of *C. rosea* genes, depending on their percentile rank of antisense sRNA counts. Percentile ranks were assigned to each gene based on its antisense sRNA counts. Genes with an antisense sRNA count of zero were not considered. The reads belong to *C. rosea* interacting with *B. cinerea* (CrBc) and F. graminearum (CrFg) or to the controls (CrCr), at the contact (C) and after contact (AC) stages of interaction.

The *C. rosea* degradome samples were sequenced to identify sRNA gene targets, 5′ noncapped degradation products of mRNA, producing between 4.2 and 10.7 million clean reads depending on the sample (Table S2). A high proportion of the reads (62% on average) from *C. rosea* and the mycohosts’ interaction was mapped to *C. rosea*. In comparison, the average counts for *B. cinerea* and F. graminearum were 31% and 32%, respectively. Mapping of degradome reads to the *C. rosea* genome showed that the majority of reads were mapped on the transcribed regions of the genome, with 54% aligning to CDS regions, 16% to 3′ UTRs, and 1.6% to 5′ UTRs. Moreover, 4.9% of the reads mapped to promoter sequences, while 13.6% were assigned to intergenic regions. A meager fraction of sequences (0.01%) was successfully mapped to known tRNAs (Fig. 5A). Degradome-based hierarchical clustering grouped degradomes from the CrBc and CrFg contact stage together. In contrast, a higher degree of similarity between the degradome from CrBc after the contact stage and self-interaction (CrCr) was found (Fig. 5B).

We analyzed the correlation between sRNAs with antisense orientation mapping and transcript cleavage to analyze sRNA-mediated transcript degradation. The genes were divided into four groups depending on their antisense sRNA counts, with each group comprising 25% of all genes and containing genes with an sRNA antisense count higher than the genes in the previous group. Then, the average degradome count was observed for each group in each interaction. Among the 25% of genes with lower antisense sRNA counts, the average degradome read count was between 160 and 270, while the same value was between 300 and 500 for the genes in the last group. This finding showed a positive correlation between mapping of antisense sRNAs to the transcripts and higher degradome count (Fig. 5C) and corroborated the use of degradome sequencing for investigating sRNA-mediated gene regulation.

### Identification of endogenous and cross-species gene targets using degradome sequencing.

**FIG 6:**
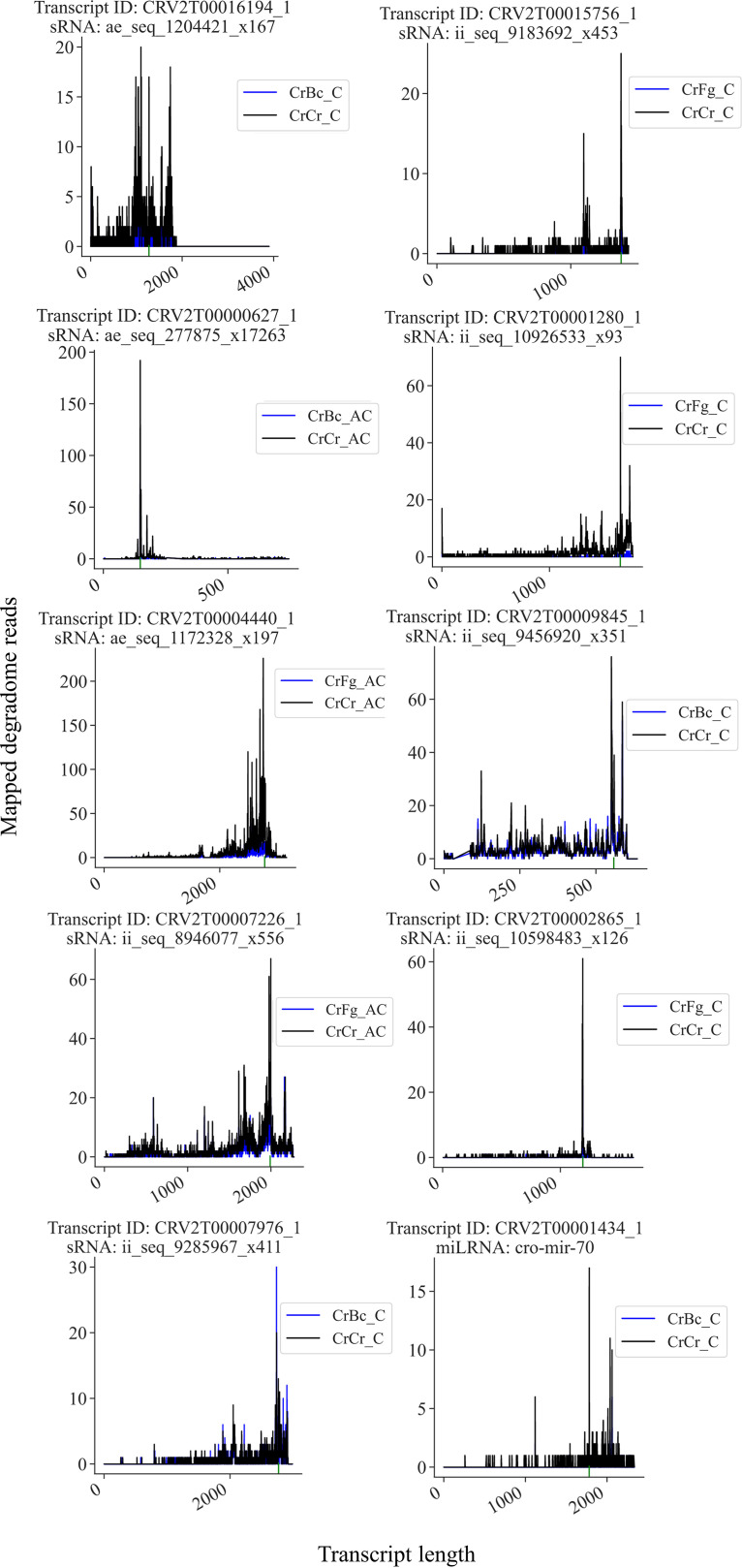
Target plots showing sRNA-mediated transcript cleavage. The mapping of degradome reads to the gene of interest are shown. Green lines under the *x* axis indicate the area of alignment between the transcript and the considered sRNA. The transcript ID of the gene and the sRNA identification number is shown for each plot.

Transcriptome-wide degradome analysis has previously been used for large-scale sRNAs target identification (13, 14). We used CleaveLand (11) on the degradome data for detection of transcripts with a higher-than-average degradome count at the point of alignment with a differentially expressed sRNA (false-discovery rate [FDR] < 0.05 and log_2_ fold change [FC] ≥ 1). Later, it was also confirmed through evaluation of differential degradation of the targets (FDR < 0.05 and log_2_[FC] ≥ 1) at the predicted point of alignment between sRNAs and transcripts. In total, we identified 201 putatively cleaved endogenous genes for 282 differentially expressed sRNAs (Data Set 3). Target plots showing comparative transcript cleaving of 10 sRNAs gene targets are presented in Fig. 6.

**FIG 7:**
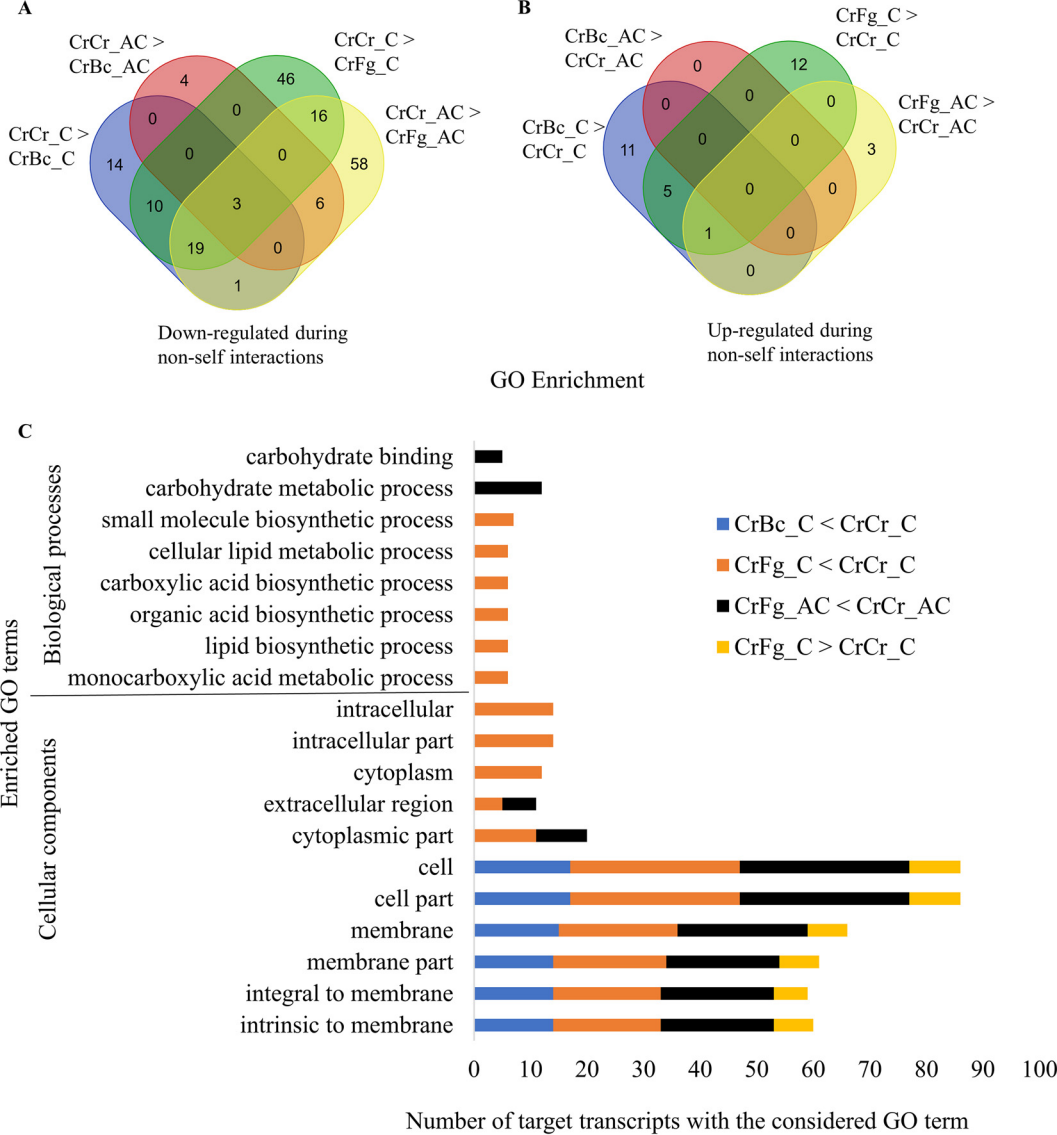
(A and B) Distribution of *C. rosea* transcripts putatively cleaved by sRNAs upregulated (A) and downregulated (B) during *C. rosea* interaction with *B. cinerea* (CrBc) and F. graminearum (CrFg) compared with self-interaction controls (CrCr), both at contact (C) and after contact (AC) stages of interactions. The cleavage by upregulated or downregulated sRNAs is confirmed by corresponding overdegradation or underdegradation at the alignment point between the transcript and the sRNA. (C) Distribution of GO terms enriched in *C. rosea* transcripts putatively cleaved by sRNA downregulated or upregulated during interactions with *B. cinerea* or F. graminearum.

We identified 47 and 13 gene targets putatively cleaved by 64 and 16 downregulated sRNAs at contact and after contact time points, respectively, during CrBc (Fig. 7A). A total of 17 transcripts, targeted by 21 upregulated sRNAs, were predicted in CrBc at contact, while no targets were predicted for the sRNA upregulated after contact (Fig. 7B). Compared to CrBc, the number of targets detected during the interaction with F. graminearum was higher. We found 197 putative gene targets for 274 downregulated sRNAs (Fig. 7A). A total of 38 gene targets were common between the two time points. For sRNAs upregulated during CrFg, we identified 22 cleaved transcripts targeted by 24 sRNAs (Fig. 7B). In summary, analyses of degradome data corroborate the mycohost and interaction stage-dependent response of *C*. *rosea* during non-self-interactions.

Additionally, we used degradome data from the interacting mycohosts and searched for potential gene targets of differentially expressed *C. rosea* sRNAs in *B. cinerea* and F. graminearum. Our result identified 43 and 91 potential gene targets in *B. cinerea* and F. graminearum with a higher-than-average degradome read count in the mapping site of a differentially expressed *C. rosea* sRNA (Data Set 4). The 43 *B. cinerea* transcripts were putatively cleaved by 40 sRNAs upregulated at the contact stage. Among the F. graminearum gene targets, 78 gene targets were putatively targeted by 90 upregulated sRNAs.

### Gene Ontology enrichment analyses.

sRNA gene targets were used for Gene Ontology (GO) term analysis to investigate biological processes, cellular components, and molecular functions enriched among sRNA gene targets during the interspecific interactions in *C. rosea*. During CrBc, GO terms GO:0016020 (membrane), GO:0016021 (integral to membrane), and GO:0031224 (intrinsic to membrane) were enriched among the targets of sRNAs downregulated at the contact stage of interaction, suggesting a role of sRNA-mediated gene silencing in regulating expression of membrane proteins, such as the phosphate permease CRV2T00001752_1 (Fig. 7C, Data Set 3). No GO terms were enriched among transcripts putatively cleaved during the postcontact stage of CrBc. Similarly, no GO terms were enriched during CrBc among the targets of upregulated sRNAs at any time point (Fig. 7C).

During CrFg, the terms GO:0032787 (monocarboxylic acid metabolic process), GO:0008610 (lipid biosynthetic process), GO:0016053 (organic acid biosynthetic process), GO:0046394 (carboxylic acid biosynthetic process), GO:0044255 (cellular lipid metabolic process), and GO:0044283 (small molecule biosynthetic process) were enriched among targets of sRNAs downregulated at contact in CrFg (Fig. 7C). Since transcript cleavage is a negative form of regulation, this points to an upregulation of primary metabolism in *C. rosea* upon contact with F. graminearum. In CrFg 24 h after contact, the enriched GO terms among targets of downregulated sRNAs were GO:0005975 (carbohydrate metabolic process) and GO:0030246 (carbohydrate binding). Regarding cellular localization, the terms GO:0005576 (extracellular region), GO:0016020 (membrane), GO:0016021 (integral to membrane), and GO:0031224 (intrinsic to membrane) were enriched among the putatively cleaved targets in CrFg both at contact and after contact (Fig. 7C), suggesting that *C. rosea* responds to F. graminearum by reducing the cleaving of transcripts that encode proteins able to interact directly with the mycohost through secretion or membrane localization. Examples of these types of proteins were the hydrophobins CRV2T00019066_1 and CRV2T00019646_1, putatively cleaved by sRNAs ii_seq_3982231_x20997, ae_seq_55370_x52182, and ae_seq_156677_x33715 at both examined time points (Data Set 3). Response to oxidative stress was also predicted to be involved in response to F. graminearum, as the catalase isozyme P (CRV2T00015756_1) was among the transcripts putatively cleaved by sRNAs (ii_seq_9183692_x453) downregulated in CrFg at contact (Data Set 3, Fig. 7C). Among the targets of upregulated sRNAs, the GO terms GO:0016020 (membrane), GO:0016021 (integral to membrane), and GO:0031224 (intrinsic to membrane) were again enriched during CrFg at contact, while no GO term was predicted at the after-contact stage (Fig. 7C). This suggests that the response to F. graminearum in *C. rosea* involves a rapid shift of membrane proteins to mediate the interaction with the mycohost.

### Identification of endogenous and cross-species gene targets of *C. rosea* milRNAs.

*C. rosea* downregulated milRNAs were predicted to cleave six endogenous genes for three downregulated milRNAs (Data Set 3). Among these, we found gene targets putatively coding for an ATP-binding cassette (ABC) transporter (CRV2T00001434_1) and five uncharacterized proteins. Upregulated milRNA cro-mir-72, on the other hand, was predicted to cleave one endogenous transcript (CRV2T00009845_1) encoding a putative transcription factor (Data Set 3).

Cross-species gene target analysis showed that cro-mir-72 was predicted to cleave the F. graminearum transcript XM_011323146.1 coding for elongation factor 3 (Data set 4). The role of elongation factor 3 in oxidative resistance is characterized in Saccharomyces cerevisiae (47).

### Mycohost-responsive milRNAs are not well conserved in *Clonostachys* spp.

**FIG 8:**
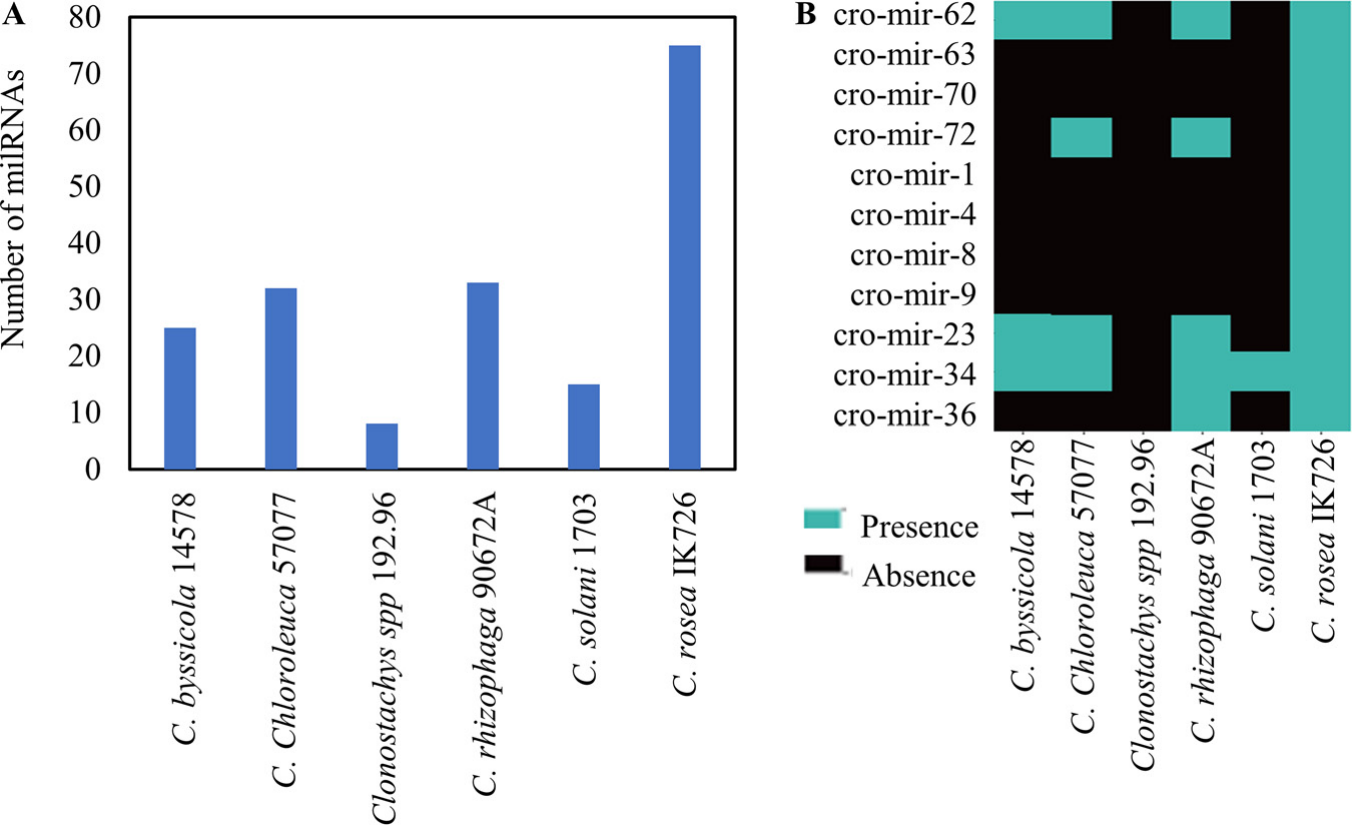
milRNA distribution in *Clonostachys* spp. (A) The number of *C. rosea* milRNAs detected in other *Clonostachys* spp. (B) Distribution of differentially produced *C. rosea* milRNAs during interactions with *B. cinerea* or F. graminearum compared with the self-interaction control (CrCr).

To evaluate milRNA conservation in the genus *Clonostachys*, subgenus *Bionectria*, we searched for milRNA precursor sequences in the genomes of five other species sequenced to date (48). Among the 74 milRNAs observed in this study and in that of Piombo et al. (41), 9 milRNAs were detected in at least 5 of the 6 *Clonostachys* spp., while 44 were detected in at least 1 more *Clonostachys* sp. (Data Set 5; Fig. 8A). However, the differentially expressed milRNAs seemed to be less conserved than average (Fig. 8B), with almost half of them detected only in *C. rosea*, while the others tended to be also observed in the more closely related C. chloroleuca, C. byssicola, and C. rhizophaga.

### Validation of degradome-based gene targets by transcriptome sequencing.

We used transcriptome data from our previous work collected at the contact stage of CrBc and CrFg interactions (41). We analyzed the expression pattern of putative gene targets identified in this study for validation. In Piombo et al. (41), transcriptomes (mRNAs and sRNAs) of *C*. *rosea* wild type (WT), the *dcl1* deletion mutant (Δ*dcl1*), and the *dcl2* deletion mutant (Δ*dcl2*) were sequenced during *in vitro* interactions with *B. cinerea* or F. graminearum. We expected sRNA-cleaved transcripts identified from degradome sequencing to be desuppressed (consequently upregulated) in the Δ*dcl1* and Δ*dcl2* strains compared to the WT, lacking sRNAs required for transcript cleavage. Using this approach, we were able to verify between 5 and 37% of the degradome-based gene targets, depending on the examined condition. Our result showed that a higher proportion of gene targets were verified for downregulated sRNAs (Fig. S4) than for upregulated sRNA gene targets. This positively correlates with sRNA expression patterns and degradome-based target prediction, as a higher proportion of sRNAs were downregulated during non-self-interactions and, consequently, a higher number of degradome-based gene targets were predicted for downregulated sRNAs. When using the data from Piombo et al. (41) to calculate the Spearman correlation between each sRNA and its targets, the average correlation value was −0.18, while the average correlation to nontarget genes was −0.12. Using a Wilcoxon rank sum test to evaluate the significance of anticorrelation between each sRNA and its predicted target, compared to the correlation to nontarget genes (49), resulted in no significant targets at a *P* value of 0.05. However, eight *C. rosea*, three *B. cinerea*, and eight F. graminearum sRNA-gene target anticorrelations were significant at a *P* value threshold of 0.1, presented in Table S3. This poor anticorrelation between sRNAs and gene targets may be attributed to differences in experimental setups between the two experiments.

### Identification of phasiRNAs.

PHASIS analysis predicted 46 phasiRNAs in *C. rosea*, belonging to 7 families (Data Set 6) originating mainly from tRNAs. Although 35 phasiRNAs were differentially expressed in *C. rosea* in at least one stage of interspecific interactions, compared with the CrCr self-interactions, no alignment between these sequences and the degradome data was found.

## DISCUSSION

*Clonostachys rosea* is a necrotrophic mycoparasite with broad range of mycohosts (24). A transcriptome study of *C. rosea* during interactions with Botrytis cinerea and F. graminearum showed both common and specific responses (39). The difference in transcriptomic response is considered to be associated with the intrinsic differences of the mycohosts, for instance, a differential composition of the cell wall and the ability to produce a different spectrum of specialized metabolites, hydrolytic enzymes, reactive oxygen species, and other virulence factors. This was confirmed by a study of Piombo et al. (41), which showed that mycohosts *B. cinerea* and F. graminearum responded differently against *C. rosea* on a transcriptional level. Genes encoding proteins involved in synthesizing and transporting specialized metabolites, including the polyketide fusarielin, and mycotoxins such as zearalenone and deoxynivalenol (DON) were upregulated in F. graminearum during interaction with *C. rosea* WT compared to a *C. rosea* Δ*dcl2* mutant strain (41). In *B*. *cinerea*, genes encoding proteins involved in cell wall biosynthesis were upregulated under the same conditions (41). Our sRNA expression and degradome tag analyses further confirms the previous finding that *C*. *rosea* can adjust its transcriptomic response, and thereby its mycoparasitic interaction mechanisms, to the identity of its mycohost. We further provide strong evidence that sRNAs plays an essential role in this regulation. On the other hand, even if seven putative phasiRNA clusters were identified, no originating milRNA was predicted, nor were any degradation products identified in the degradome data. This suggests that phasiRNAs are not present in *C. rosea* or that they are difficult to predict with plant-based software.

The common response against both mycohosts includes enrichment of degradome tags from genes coding for membrane transporters known for their important role in antagonistic interactions (36, 39, 48, 50, 51). The result confirms that the regulation of genes coding for membrane transporters is an important common response of *C*. *rosea* during interspecific fungal interactions (38, 39, 41) and that sRNAs mediate this process. Additionally, enrichment of genes encoding membrane proteins among the sRNA targets points to a drastic replacement of the protein part of the membrane during non-self-interactions of *C. rosea*. The degradome tags of genes putatively associated with biosynthetic processes, including lipid biosynthesis, were enriched specifically against F. graminearum. This suggests that *C. rosea* desuppresses the expression of lipid biosynthesis genes, possibly to maintain the functionality of the plasma membrane that may be a target for toxic metabolites produced by F. graminearum. It is known that Fusarium spp. produce toxic compounds for interference competition (41, 42). For instance, the mycotoxin DON is shown to downregulate the expression of chitinase genes associated with the mycoparasitic attack in *Trichoderma* spp. (52). This fits well with the gene expression profile of genes related to specialized metabolite biosynthesis, including DON. DON-biosynthesis genes were downregulated when F. graminearum grew in contact with a *C. rosea* Δ*dcl2* mutant with diminished biocontrol capabilities, in respect to F. graminearum interacting with *C. rosea* WT, suggesting that this mycotoxin is needed to overcome the antagonistic activity of *C. rosea* (41). Similar results were reported previously, where expression of genes coding Kp4 killer toxins were upregulated in F. graminearum during the interaction with the mycoparasitic fungus Trichoderma gamsii (42).

Another plausible explanation of the differential response is related to the degree of antagonistic ability of the mycohosts. The fungal prey is not passively growing toward the mycoparasite. On the contrary, it can actively launch a counterattack involving fungal cell wall-degrading enzymes, toxic specialized metabolites, and production of reactive oxygen species (53). This is also reflected by the result from the *in vitro* dual culture plate confrontation assay, which revealed a growth rate inhibition of *C*. *rosea* during the interaction with F. graminearum, but not with *B. cinerea*. The overgrowth rate of *C*. *rosea* on *B*. *cinerea* mycelia was similar to the growth rate in the noninteraction control, suggesting that *C. rosea* can quickly overcome the counterattack by *B*. *cinerea*. This is verified by the transcriptome-wide comparative degradome analysis between two interaction stages, which showed how the transcript cleavage pattern 24 h after contact with *B. cinerea* was more similar to the *C. rosea* self-interaction control than to the *B. cinerea* contact stage. This suggests that *C. rosea* quickly overcame *B. cinerea* and that the transcript levels were already going back to normal 24 h after contact, while the contact and 24-h postcontact stages with F. graminearum remained very similar to one another. Coexpression analysis further highlights the mycohost- and interaction stage-dependent responses of *C*. *rosea*, with modules 10 and 11 showing an expression in CrBc at after contact more similar to that of the CrCr control than to that of CrBc at contact. This also emphasizes that the mycelial contact stage is crucial for non-self-interactions for modulation of the mycoparasitic regulatory network in *C. rosea*. Zapparata et al. (42) also showed extensive communication between the mycoparasite *T. gamsii* and F. graminearum resulting in transcriptomic modifications in both fungi, even before physical contact.

Another interesting finding is that the number of downregulated sRNAs is greater than the number of upregulated ones, and the number of putative targeted transcripts followed a similar pattern. Mechanistically, this suggests that genes encoding proteins involved in the response of *C. rosea* to mycohosts are constantly suppressed by sRNA-mediated RNAi when the mycohosts are absent. However, in the presence of mycohosts, *C. rosea* reduces the production of sRNAs, thereby inducing the consequent desuppression (upregulation) of the gene targets. Among the targets of commonly downregulated (against both mycohosts) sRNAs, we find transcripts coding for secreted proteins, such as the two hydrophobins CRV2T00019066_1 and CRV2T00019646_1, belonging to a class with a proven role in *C. rosea* root colonization (34). Their upregulation during the interaction with pathogens suggests a link between the antagonistic role of *C. rosea* and its ability to interact with plants (24).

The role of sRNAs in cross-kingdom RNAi is established (19, 20). To investigate sRNA-mediated cross-species RNAi in mycoparasitic interactions, mycohost transcripts were also predicted as possible targets (cross-species gene targets) by *C. rosea* sRNAs and milRNAs. Among these, we found many genes encoding proteins involved in basal cellular homeostasis, suggesting that *C. rosea* uses RNAi to disrupt core metabolic pathways of its mycohosts. In *B. cinerea*, the targets include malate dehydrogenase BcCMDH1, necessary for mitochondrial function (54), and BcCDC48, involved in cell division control (55). In F. graminearum, putative targets include elongation factors 1 and 2, involved in the enzymatic delivery of aminoacyl tRNAs to the ribosome (56, 57), and the nascent polypeptide-associated complex subunit alpha, whose role is to promote interactions between ribosomes and the mitochondrial surface (58).The milRNA cro-mir-9, upregulated during contact with F. graminearum, also had two putative cross-regulated targets in Piombo et al. (41), encoding a zinc-binding protein (XM_011320613.1) and a Sec1 family vesicle transport protein (XM_011329717.1). This family is essential for SNARE (soluble *N*-ethylmaleimide-sensitive attachment protein receptor)-mediated membrane fusion in eukaryotes (59) and is therefore involved in a vast array of biological processes, including virulence in C. neoformans (60). Moreover, cro-mir-4 showed upregulation at contact in CrBc, and this milRNA was suggested to have several putative cross-regulated targets in a previous study (41). These include the GT2 chitin synthase BcCHSIV and a homolog of the pp-z protein (XM_024693385.1). This protein is involved in oxidative stress response and virulence in Candida albicans (61), and oxidative stress is often present at mycoparasitic interaction sites (53). Another putative gene target was encoding the vacuolar sorting protein BcVPS27, whose homolog deletion in *B. cinerea* causes a reduction in growth rate, aerial hypha formation, and hydrophobicity, as well as increasing sensitivity to cell wall-damaging agents and to osmotic stresses (62). Furthermore, several genes involved in gene expression regulation are also predicted to be targeted by cro-mir-4, suggesting that *C. rosea* can affect *B. cinerea* gene regulation at a higher level to carry out its antagonistic activity. These include the chromatin binding factor gene *Bcyta7* (63) and the transcription factor gene *Bcltf3*, necessary for conidiogenesis (64). It must be stressed that additional evidence is needed to validate the cross-regulation events.

### Conclusions.

The presented work increases our understanding of the mechanisms involved in interspecific fungal interactions, with important implications for the use of fungi as biological control agents. We show that several *C. rosea* sRNAs are downregulated during interactions with *B. cinerea* and F. graminearum. Consequently, their putative gene targets are predicted to be upregulated (desuppressed), suggesting a role of sRNA-mediated regulation of mycoparasitism in *C. rosea.* These putative *C. rosea* sRNA-regulated transcripts are often coding for membrane transporters or secreted proteins. We further show that the response of *C*. *rosea* toward *B. cinerea* and F. graminearum depends on mycohost identity and interaction stage and that sRNAs are part of the regulatory mechanism. This is important, as it shows that *C. rosea* can adapt its transcriptional response, and thereby its interaction mechanisms, based on the identity of the mycohost. Finally, our data strongly suggest a role of cross-species RNAi in mycoparasitism, representing a novel mechanism in biocontrol interactions. This can find important applied uses in spray-induced gene silencing for improved efficacy of biocontrol applications.

## MATERIALS AND METHODS

### Experimental setup for sRNA and degradome sequencing.

*Clonostachys rosea* strain IK726, *B. cinerea* strain B05.10, and F. graminearum strain PH-1 were used in the study. An *in vitro* dual-culture experiment was performed for sRNA and degradome sequencing during the interaction, following previously described procedures (35). In brief, an agar plug of *C*. *rosea* mycelium was inoculated at the edge of a 9-cm-diameter potato dextrose agar (PDA; Merck, Kenilworth, NJ) petri plate covered with a Durapore membrane filter (Merck, Kenilworth, NJ) for an easy harvest of mycelia. The mycohost fungi *B. cinerea* and F. graminearum were inoculated at opposite sides of the plate (37). The mycelial front (5 mm) of *C*. *rosea* was harvested together with the mycelial front (5 mm) of *B*. *cinerea* (CrBc) or F. graminearum (CrFg) at the hyphal contact stage (early physical contact between the mycelia) and at 24-h post-hyphal contact stage of interactions. Mycelium harvested at the same stage from *C*. *rosea* confronted with *C*. *rosea* (CrCr) was used as a control treatment. The experiment was performed in four biological replicates.

### RNA extraction and sequencing.

Total RNA extraction was performed using the mirVana miRNA isolation kit following the manufacturer’s protocol (Invitrogen, Waltham, MA). The RNA quality was analyzed using a 2100 Bioanalyzer instrument (Agilent Technologies, Santa Clara, CA), and RNA concentration was quantified using a Qubit fluorometer (Life Technologies, Carlsbad, CA). For sRNA sequencing, the total RNA was sent for library preparation and paired-end sRNA sequencing at the National Genomics Infrastructure (NGI) Stockholm, Sweden. The sRNA library was generated using a TruSeq sRNA kit (Illumina, San Diego, CA) and sequenced on one NovaSeq SP flow cell with 2 × 50-bp reads using Illumina NovaSeq 6000 equipment at NGI Stockholm. The Bcl to FASTQ conversion was performed using bcl2fastq v.2.19.1.403 from the CASAVA software suite. The quality scale used is Sanger/phred33/Illumina v.1.8+.

### Functional annotation of genomes.

Blast2GO v.5.2.5 (65) and InterProScan v.5.46-81.0 (66) were used to annotate the proteomes of *C. rosea* strain IK726 (BioProject no. PRJEB4200), *B. cinerea* strain B05.10 (ASM14353v4), and F. graminearum strain PH-1 (ASM24013v3). Putative CAZymes were identified through the dbCAN2 meta server (67).

### sRNA sequence analysis.

**FIG 9:**
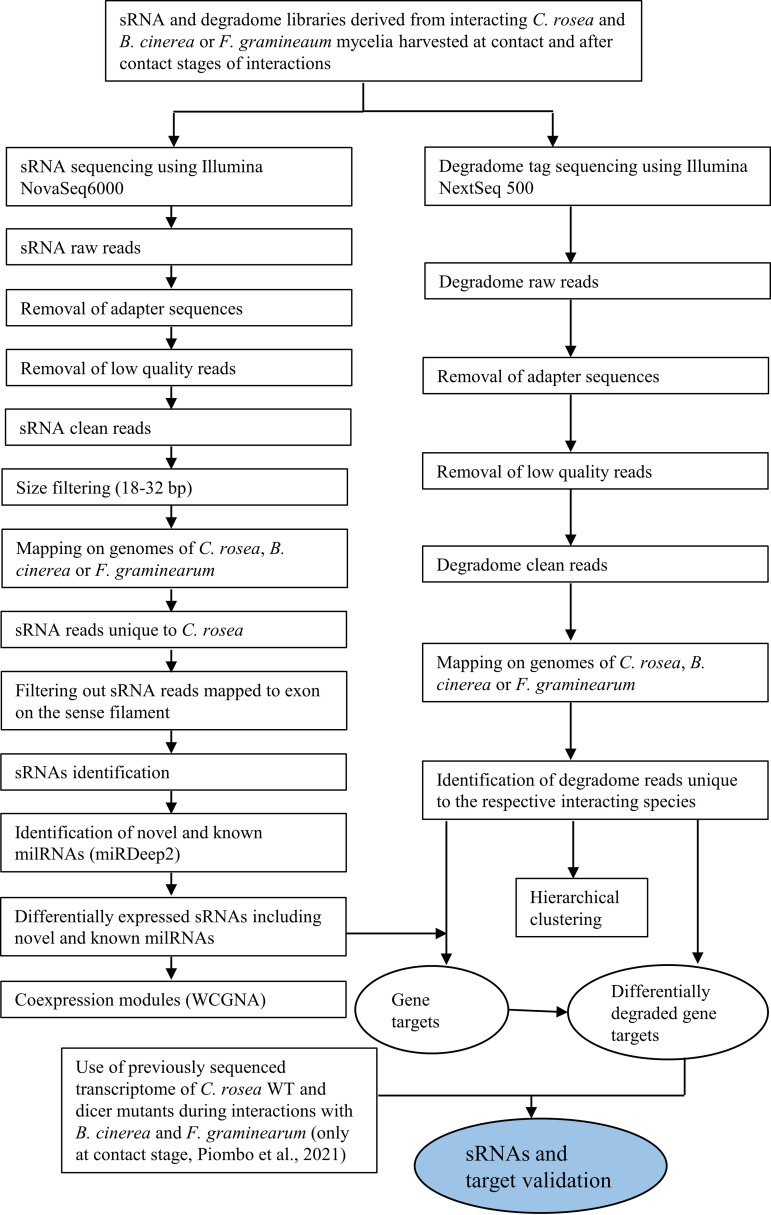
Flowchart of sRNAs and degradome sequence analyses showing different steps in identifying *C. rosea* sRNAs (including milRNAs) and their potential endogenous and cross-species gene targets.

All the analysis done on sRNAs is summarize in Fig. 9. Raw reads received after sequencing were trimmed with cutadapt v.2.8 (68), setting 16 bp as the minimum allowed length, and quality was checked with FastQC v.0.11.9 (https://www.bioinformatics.babraham.ac.uk/projects/fastqc/) before and after the trimming. Reads shorter than 18 bp or longer than 32 bp were removed, and the cleaned reads were then mapped to the genomes of *C*. *rosea* strain IK726 (GCA_902827195.2) (69), *B*. *cinerea* strain B05.10 (GCF_000143535.2) (70), and F. graminearum strain PH-1 (GCF_000240135.3) (71) using STAR v.2.7.5c (72) with the following parameters: STAR –outFilterMultimapNmax 20 –outFilterMismatchNoverLmax 0.05 –outFilterMatchNmin 16 –outFilterScoreMinOverLread 0 –outFilterMatchNminOverLread 0 –alignIntronMax 1 –alignEndsType EndToEnd. To exclude the sRNA sequences originating from the mycohosts, the reads mapping exclusively to the *C*. *rosea* genome were retained and were used for further analysis. Sense and antisense sRNAs mapping to intergenic regions and introns, as well as antisense sRNAs mapping to exons, were detected, and DESeq2 v.1.28.1 (73) was used for differential sRNA expression analysis at the cutoff of log_2_ FC of 1 and *P* value (adj) = 0.05. Moreover, milRNAs were predicted with miRDeep2 with default parameters (46). Both known and novel milRNAs were predicted, and DESeq2 v.1.28.1 (73) was used to determine differential expression.

To ensure the novelty of newly detected milRNAs, they were compared with the fungal milRNAs identified in several other studies, plus all the fungal milRNAs available in RNAcentral, using the BLAST algorithm with 95% minimum identity (13, 16, 17, 74–78).

### Coexpression analysis.

Normalized expression values were obtained for sRNAs by using DESeq2 (73). The values of differentially expressed sRNAs were used to perform a coexpression analysis with WGCNA (79) using a soft-thresholding power of 6. The function binarizeCategoricalVariable was used to convert the mycohost and interaction stage categorical variables into numerical ones, and the Spearman correlation was calculated between them and the module eigengenes.

### MilRNA target prediction.

The UTR regions of *B. cinerea*, F. graminearum, and *C. rosea* genes were determined with add_utrs_to_gff (https://github.com/dpryan79/Answers/tree/master/bioinfoSE_3181), and they were used for target prediction of DCL2-dependent milRNAs with animal-based tools PITA, Miranda, TargetSpy, and simple seed analysis within the sRNAtoolbox (80). The sRNA toolbox was also used to run the plant-based target predictors PsRobot and TAPIR (81, 82), while TargetFinder and psRNATarget were used independently (83, 84). Target-milRNA couples predicted by at least 3 animal-based tools or 2 plant-based ones were considered in the following analyses.

### Degradome sequencing and analysis.

To sequence uncapped 5′ ends from poly-adenylated RNA (degradome-seq), total RNA isolated from the above-mentioned samples was used. For degradome sequencing, total RNAs, after DNase treatment, were sent to GenXPro GmbH (Frankfurt, Germany). The degradome libraries were generated by GenXPro using the MACE-Seq kit (massive analysis of cDNA ends) (GenXPro GmbH) and the TrueQuant sRNA-Seq kit (GenXPro GmbH). Briefly, mRNA was captured using Dynabeads Oligo(dT) (Invitrogen, Waltham, MA). The 5′ adapter from the TrueQuant sRNA-Seq kit (GenXPro GmbH) was ligated to the uncapped 5′ ends of the poly-A transcripts. Reverse transcription and PCR were performed according to the TrueQuant sRNA-Seq kit manual, and the degradome Tags were sequenced on an Illumina Next 500 instrument. Reads were trimmed with bbduk v.38.86 (85) with the following options: bbduk.sh in1=read1.fastq in2=read2.fastq out1=read1_clean.fastq out2=read2_clean.fastq ref=.fa ktrim=r k=23 mink=11 hdist=1 tpe tbo qtrim=r trimq=10. The analysis of degradome data is summarized in Fig. 9.

Successful cleaning and adapter removal were verified with FastQC v.0.11.9 (https://www.bioinformatics.babraham.ac.uk/projects/fastqc/). Since all the samples represented the interaction of two organisms, the genome of *C. rosea* was concatenated with the one of either *B. cinerea* or F. graminearum, creating two combined genome files (CrFg and CrBc), and the same was done with the annotations in gff format. Degradome reads from *C. rosea*-*B. cinerea* interactions were aligned to the CrBc genome, while reads from *C. rosea*-F. graminearum interactions were aligned to the CrFg equivalent. Multimapping reads were removed from the analysis. The chosen aligner was STAR v.2.7.5c (86), with default options, and the count tables were then generated through FeatureCounts v.2.0.1 (87). Only sense reads were considered for mapping to known features, while both sense and antisense reads were considered when mapping to intergenic regions. Variance stabilizing transformation was applied to visualize the hierarchical clustering with the R pheatmap package (88) following the steps presented in the Bioconductor DESeq2 tutorial (http://bioconductor.org/packages/devel/bioc/vignettes/DESeq2/inst/doc/DESeq2.html).

Differentially expressed sRNAs mapping to intron or intergenic regions, and antisense sRNAs mapping to exons, were used together with degradome reads to predict which genes were regulated through RNA silencing, by using the CleaveLand program v.4.5 (11). Only genes flagged as category 2 or better degradation targets in all replicates were retained as putative targets of RNA silencing. Category 2 means that a higher than average degradome read count was present at the mapping site of the considered sRNA (https://github.com/MikeAxtell/CleaveLand4). After this step, we estimated differential degradation at the point of alignment between each sRNA and its target, using DESeq2 with default parameters and, as input, only counts of degradome reads mapping to the point of alignment predicted by CleaveLand. We retained only the results in which the differential degradation and the differential sRNA expression showed correlation (for example, underdegradation in targets of downregulated sRNAs). Furthermore, the expression level of transcripts putatively cleaved by sRNAs was checked using the data from Piombo et al. (41), verifying how many of the putative targets were upregulated in *C. rosea* Dicer deletion mutants, devoid of a functional Dicer-dependent RNA silencing system. Additionally, the data from Piombo et al. (41) were also used to test the Spearman correlation between each sRNA and its putative targets, using a minimum of 50 reads as a cutoff for both transcripts and sRNAs. The Spearman correlation of each sRNA-target couple was compared with the same correlation measured between the sRNA and a random sample of 100 genes, using the Wilcoxon rank sum test as done in a previous study (49). *Clonostachys rosea* random genes were used to evaluate *C. rosea* gene targets, while genes of *B. cinerea* and F. graminearum were used to evaluate their respective putative targets. All genes used for validation were covered by at least 1,000 reads. An *ad hoc* python script with the pandas and SciPy modules was used to perform this operation (89, 90).

Enrichment in GO terms in the set of genes targeted by differentially expressed sRNAs was determined through Fisher tests performed with agriGO (91) using the Yekutieli multitest adjustment method and an FDR threshold of 0.05.

### PhasiRNA prediction.

PhasiRNAs in the data set were predicted with PHASIS v.3 (92) setting minimal abundance to 10, and differential expression was analyzed with DESeq2 v.3.13 (73). Target prediction was carried out with TargetFinder, PsRobot, TAPIR, and psRNATarget, and only targets copredicted by at least 2 tools were considered (80–84).

### MilRNAs detection in other *Clonostachys* spp.

The presence of novel and known milRNAs was investigated in the genomes of *C. byssicola* CBS 245.78, *C. chloroleuca* CBS 570.77, *Clonostachys* sp. strain CBS 192.96, *C. rhizophaga* CBS 906.72A, and Clonostachys solani 1703 (48). The analysis was done through BLAST with the option –task blastn-short, using a 95% threshold in both identity and query coverage for milRNA mature sequences and 90% for the entire precursor sequences.

### Stem-loop RT-qPCR.

Stem-loop RT-qPCR primers were designed for specific miRNAs (93) (Table 2). Prior to reverse transcription, the stem-loop primers were denatured by incubating them at 65°C for 5 min and transferring them to ice immediately. Reverse transcription was carried out by adding the denatured stem-loop primer (final concentration [conc.], 0.05 μM) to the following components: deoxynucleoside triphosphate (dNTP’ final conc., 0.25 mM), 10× SuperScript [SS] III buffer (final conc., 1×), dithiothreitol (final conc., 10 mM), RNaseOUT RNA inhibitor (final conc., 0.2 U/μL), SS III reverse transcriptase enzyme (final conc., 2.5 U/μL; Invitrogen, Waltham, MA), and reverse primer for *C. rosea* reference gene actin (*act*) (final conc., 0.25 μM). RNA (10 ng) from the respective control and treatment samples was used as the template, and the reaction volume was made up to 20 μL using nuclease-free water. In a thermal cycler, the following reaction conditions were used: 16°C incubation for 30 min and 60 cycles consisting of 30°C for 30 s, 42°C for 30 s, and 50°C for 1 s. The reaction was then terminated by enzyme inactivation at 85°C for 5 min.

RT-qPCR was performed with a DyNAmo Flash SYBR green kit (Thermo Fisher Scientific, Waltham, MA) to validate the relative miRNA expression. *Clonostachys rosea* actin was included as a reference gene for normalization. The Livak method (2^–ΔΔ^*^CT^*) was employed for quantification of gene expression (94).

### Data availability.

The sequencing data generated and analyzed in this work have been deposited in the European Nucleotide Archive (ENA) at the European Molecular Biology Laboratory (EMBL) under the BioProject accession number PRJEB51338. This project contains both degradome and sRNA sequencing data for all samples.
