# Supplementary material for: Comparative Small RNA and Degradome Sequencing Provides Insights into Antagonistic Interactions in the Biocontrol Fungus Clonostachys rosea
Source: Appl Environ Microbiol. 2022 Jun 13;88(13):e00643-22. doi: 10.1128/aem.00643-22 (PMC9275246; doi:10.1128/aem.00643-22)
Supplement: Supplemental file 7 — Tables S1 to S3, Fig. S1 to S4, and descriptions of Data Sets S1 to S6. Download aem.00643-22-s0007.pdf, PDF file, 0.3 MB [file aem.00643-22-s0007.pdf]

Supplementary materials

Supplemental table S1: Summary of sRNA sequencing.

Supplemental table S2: Summary of degradome sequencing and percentage of degradome reads mapping to genome features.

Supplemental table S3: Differentially expressed sRNAs in *C. rosea* during non-self-interactions with *B. cinerea* or *F. graminearium* compared to self-interaction control, and gene targets significantly anticorrelated with them.

Dataset 1: Differentially expressed sRNAs in *Clonostachys rosea* during interaction with *B. cinerea* (CrBc) and *F. graminearum* (CrFg) compared with CrCr control, at contact (C) and after contact (AC) stages of interactions.

Dataset 2: Sequence, abundance, origin and genome coordinates of the milRNAs detected in this study.

Dataset 3: Endogenous transcripts (endogenous gene targets) putatively cleaved by differentially expressed sRNAs.

Dataset 4: Putative cross-species gene targets in *B. cinerea* and *F. graminearum* putatively cleaved by differentially expressed *C. rosea* sRNAs.

Dataset 5: Distribution of the precursor and mature sequences of *C. rosea* novel and known milRNAs in *Clonostachys* spp.

Dataset 6: Sequence, origin and coordinates of the phasiRNA families detected in this study.

Supplemental figure S1: Schematic illustration of *in vitro*-dual culture plate confrontation assay used in this study for sRNAs and degradome sequencing experiment. (**A**) contact stage, (**B**) after contact stage of interactions.

Supplemental figure S2: Growth rate of *C. rosea*, *B. cinerea* and *F. graminearum* on PDA. **(A**) Growth rate of *C. rosea* during non-interaction control (Cr), self-interaction (CrCr) and non-self-interaction with *B. cinerea* (CrBc) and *F. graminearum* (CrFg). (**B**) The growth rate of *B. cinerea* during non-interaction control (Bc), self-interaction (BcBc) and non-self-interaction with *C. rosea* (CrBc). (**C**) The growth rate of *F. graminearum* during non-interaction control (Fg), self-interaction (FgFg) and non-self-interaction with *C. rosea* (CrFg). Agar plugs were inoculated on opposite sides in 9 cm diameter agar plates and incubated at 25°C. Growth rates of *C. rosea*, *B. cinerea* and *F. graminearum* were recorded daily two days post inoculation (dpi) until the mycelial contact. Due to the slower growth rate of *C. rosea*, *B.cinerea*, or *F. graminearum* were inoculated 7dpi in non-self-interaction experiments.

Supplemental figure S3: Heatmap showing the RPKM values obtained by mapping differentially expressed sRNAs to their scaffolds of origin. Only differentially represented scaffolds with a minimum RPM count of 70 are presented.

Supplemental figure S4: Validation degradome based gene targets by transcriptome sequencing. Percentage of genes, putatively cleaved by differentially expressed sRNAs in this study, were also up-regulated in the Δ*dcl2* mutant during interaction with *B. cinerea* and *F.graminearum* in Piombo *et al.* There was no degradome-based gene target to validate up-regulated sRNA at afar contact stage of CrBc (CrBc_AC_up-regulated).

Table S1: Summary of sRNA sequencing.

Treatment	Read counts in million			
	Total reads	Trimmed reads	Reads unique to *C. rosea*	Unique reads in percent
CrCr C (A)	60.58	58.10		
CrCr C (B)	57.34	53.00		
CrCr C (C)	37.94	34.50		
CrBc C (A)	39.71	36.4	10.9	29.945
CrBc C (B)	47.44	43.4	13.6	31.336
CrBc C (C)	50.48	46.8	14.1	30.128
CrBc C (D)	53.6	50.6	13.67	27.016
CrFg C (A)	43.29	38.1	10.89	28.583
CrFg C (B)	52.57	47.4	14.82	31.266
CrFg C (C)	45.09	40.9	11.22	27.433
CrFg C (D)	45.41	40.4	11.63	28.787
CrCr AC (A)	25.35	24.1		
CrCr AC (B)	52.03	48.7		
CrCr AC (C)	57.31	54.8		
CrCr AC (D)	44.44	41.6		
CrBc AC (A)	48.37	45.2	13.54	29.956
CrBc AC (B)	44.8	42	12.52	29.810
CrBc AC (C)	46.65	43.3	12.83	29.630
CrBc AC (D)	42.55	39.8	11.46	28.794
CrFg AC (A)	32.74	28.5	8.27	29.018
CrFg AC (B)	46.7	41.9	12.02	28.687
CrFg AC (C)	35.32	31.2	9.18	29.423
CrFg AC (D)	42.32	38.1	11.41	29.948
**Total**	**1052.03**	**968.8**	**192.06**	

CrCr, *Clonostachys rosea* interaction with self (control treatment); CrBc, *C. rosea* interaction with *Botrytis cinerea*; CrFg, *C. rosea* interaction with *Fusarium graminearum*. C, contact stage, AC, after contact stage. The experiment was performed in four biological replicates (A-D) with the exception of CrCr C, that had 3 replicates.

Table S2: Summary of degradome sequencing and degradome reads mapping (in percent) to genome features**.**

	**CrCr C**	**CrCr AC**	**CrBc C**	**CrBc AC**	**CrFg C**	**CrFg AC**	**Average**
CDS	53.30	53.72	53.40	54.10	54.40	57.81	54.46
3'UTR	13.13	13.75	20.29	16.13	15.43	15.07	15.63
5'UTR	1.25	1.24	2.15	1.93	1.47	1.41	1.57
Promoter	5.54	5.51	3.82	4.33	5.05	4.90	4.86
Intron	0.97	0.83	0.60	0.72	0.70	0.76	0.76
tRNA	0.01	0.01	0.01	0.01	0.01	0.01	0.01
Intergenic	16.33	15.69	10.91	13.76	12.76	12.02	13.58
Mapped on *C. rosea*	93.65	93.83	55.99	68.59	63.80	59.74	72.60
Mapped on Mycohosts	NA	NA	37.67	24.92	28.81	34.33	31.43
Total mapped	93.65	93.83	93.66	93.52	92.61	94.06	93.56
Clean reads	6588491	6751578	6398727	6807665	6162364	6601681	6551751

CrBc, *C. rosea* interaction with *Botrytis cinerea*; CrFg, *C. rosea* interaction with *Fusarium graminearum*. C, contact stage, AC, After contact stage.

Table S3: Differentially expressed sRNAs in *C. rosea* during non-self-interactions with *B. cinerea* or *F. graminearum* compared to self-interaction control, and gene targets significantly anticorrelated with them.

sRNA or milRNA	Transcript	Spearman correlation	Average Spearman correlation	Wilcoxon rank sum test pvalue	Log2FC sRNA expression*				Annotation
					CrBc_C	CrBc_AC	CrFg_C	CrFg_AC	
***C. rosea* targets**									
cro-mir-1	CRV2T00005300_1	-0.65	-0.23	0.0997	**-2.08**	**-1.14**	**-1.77**	**-1.76**	Uncharacterized protein
ii_seq_8130286_x1148	CRV2T00006986_1	-0.2	0.31	0.0863	**-1.82**	**-1.49**	**-1.83**	**-2.10**	Uncharacterized protein
ii_seq_9915934_x236	CRV2T00011384_1	-0.55	-0.11	0.0863	**-3.84**	-1.23	**-4.42**	**-2.61**	Uncharacterized protein
ii_seq_3982231_x20997	CRV2T00019066_1	-0.73	0.02	0.0863	**-3.46**	-1.21	**-3.82**	**-2.36**	Cerato-ulmin hydrophobin
ii_seq_10817621_x103	CRV2T00012492_1	-0.45	0.28	0.0863	-2.22	-1.48	-1.11	**-2.25**	Peptidase M35 family
ae_seq_55370_x52182	CRV2T00019646_1	-0.78	-0.03	0.0863	**-4.73**	**-1.90**	**-3.76**	**-2.79**	Cerato-ulmin hydrophobin
ae_seq_156677_x33715	CRV2T00019646_1	-0.81	-0.06	0.0863	**-4.21**	**-1.74**	**-3.38**	**-2.98**	Cerato-ulmin hydrophobin
ae_seq_1232116_x142	CRV2T00001752_1	-0.65	-0.11	0.0928	**-2.60**	-0.53	-1.51	-1.98	Phosphate-repressible phosphate permease
***B. cinerea* targets**									
ii_seq_10694462_x116	XM_001553799.2	-0.86	-0.25	0.0863	**2.79**	-0.14	1.64	-0.27	Malate dehydrogenase Bcmdh1
ii_seq_9510169_x336	XM_001553073.2	-0.9	-0.32	0.0863	**2.71**	0.27	1.71	-0.39	Putative gtp-binding protein
ii_seq_9879814_x242	XM_001553900.2	-0.67	-0.23	0.0997	**3.06**	1.58	**5.17**	**4.53**	Cell division control protein Bccdc48
***F. graminearum* targets**									
ii_seq_10383433_x154	XM_011328867.1	-0.49	0.11	0.0863	1.92	0.49	**2.37**	0.03	Elongation factor 1-gamma 1
ii_seq_10387573_x153	XM_011320768.1	-0.73	-0.37	0.0863	1.58	-0.81	**2.51**	0.73	Uncharacterized protein
ii_seq_9396274_x372	XM_011320768.1	-0.88	-0.41	0.0863	**2.01**	0.46	**2.21**	-0.21	Uncharacterized protein
ii_seq_10593569_x126	XM_011321707.1	-0.76	-0.39	0.0895	**2.72**	1.32	2.41	**2.63**	Transaldolase
ii_seq_10718854_x113	XM_011329540.1	-0.59	0.15	0.0863	1.60	-1.38	**3.34**	0.80	Putative RNA-binding protein
ii_seq_11023229_x85	XM_011321890.1	-0.78	-0.18	0.0863	2.77	NA	**3.15**	**2.94**	Nascent polypeptide-associated complex subunit alpha
ae_seq_1131453_x257	XM_011327288.1	-0.74	-0.38	0.0928	**2.19**	0.50	**2.56**	0.66	Uncharacterized protein
ae_seq_1131453_x257	XM_011329835.1	-0.76	-0.38	0.0928	**2.19**	0.50	**2.56**	0.66	Elongation factor 2

Cr, *C. rosea*; Bc, *B. cinerea*; Fg, *F. graminearum*; C, contact stage; AC, After contact stage.
*Significant differences (FDR 0.05 and log2fold change > 1) are highlighted in boldface.

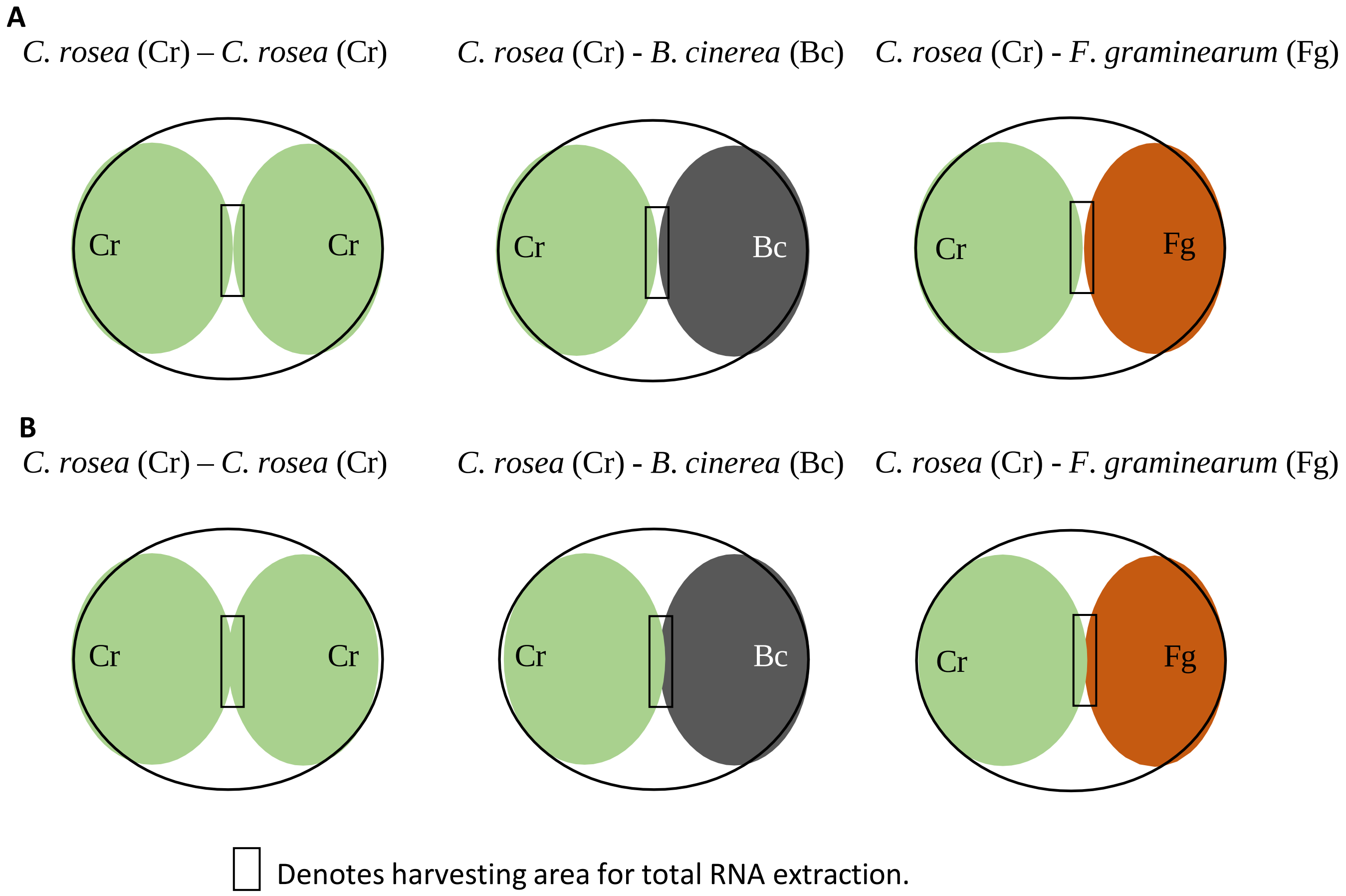

Supplemental figure S1: Schematic illustration of *in vitro*-dual culture plate confrontation assay used in this study for sRNAs and degradome sequencing experiment. (A) contact stage, (B) after contact stage of interactions.

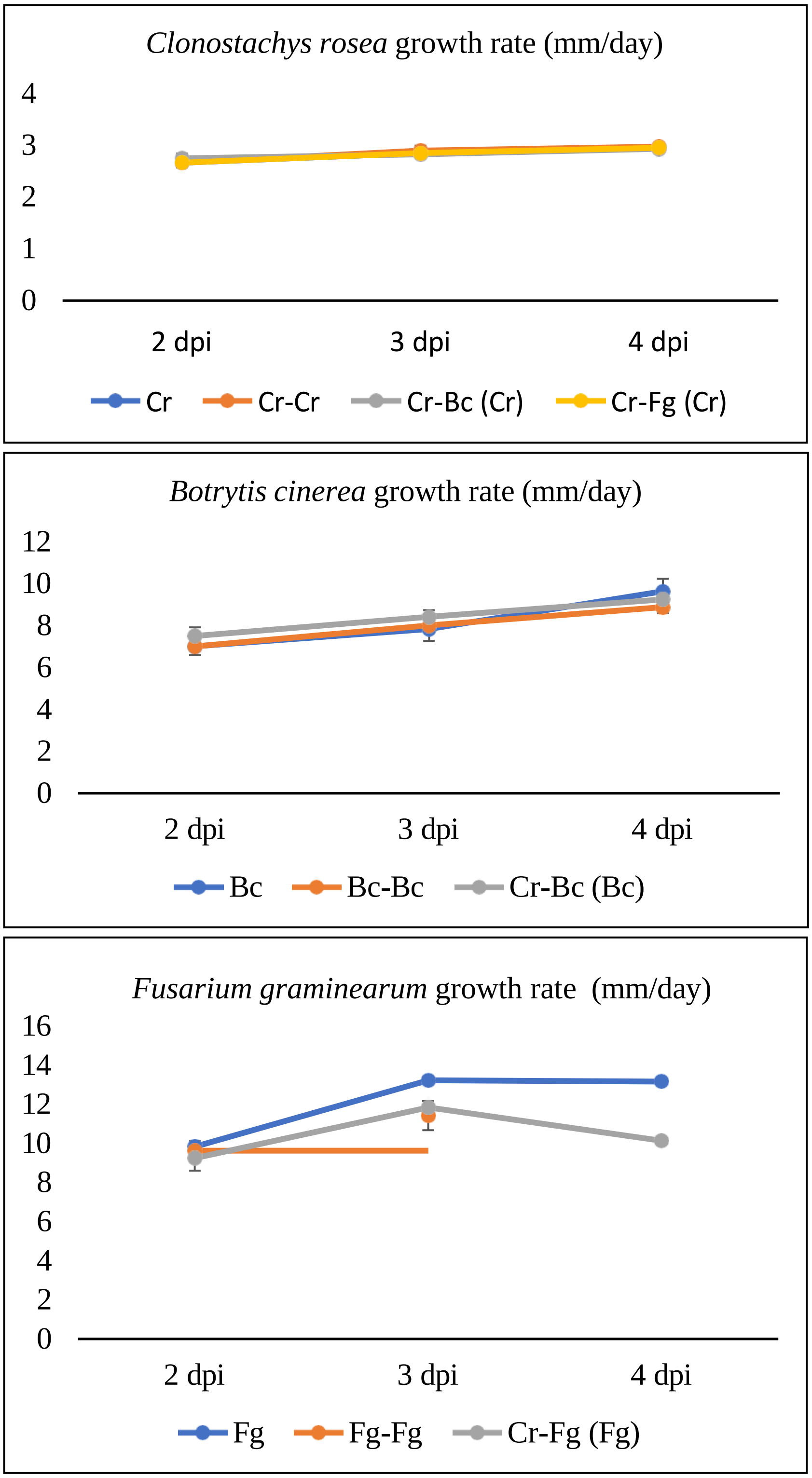

Supplemental figure S2: Growth rate of *C. rosea*, *B. cinerea*and and *F. graminearum* on PDA. **A)** Growth rate of *C. rosea* during non-interaction control (Cr), self-interaction (CrCr) and non-self interaction with *B. cinerea* (CrBc) and *F. graminearum* (CrFg). **B**) Growth rate of *B. cinerea* during non-interaction control (Bc), self-interaction (BcBc) and non-self-interaction with *C. rosea* (CrBc). **C**) Growth rate of *F. graminearum* during non-interaction control (Fg), self interaction (FgFg) and non-self interaction with *C. rosea* (Cr-Fg). Agar plugs were inoculated on opposite sides in 9 cm diameter agar plates and incubated at 25°C. Growth rates of *C. rosea*, *B. cinerea* and *F. graminearum* were recorded daily two days post inoculation (dpi) until the mycelial contact. Due to slower growth rate of *C. rosea*, *B. cinerea*or or *F. graminearum* were inoculated 7dpi in non-self interaction experiment.

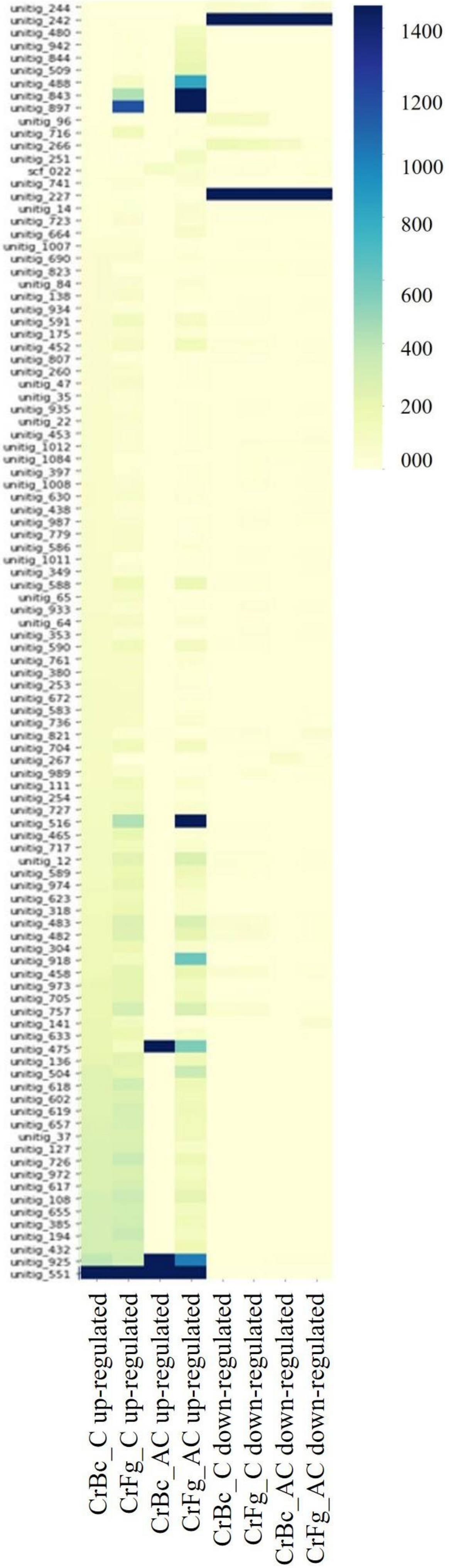

Supplementary figure S3: Heatmap showing the RPKM values obtained by mapping differentially expressed sRNAs to their scaffolds of origin. Only differentially represented scaffolds with a minimum RPM count of 70 are presented.

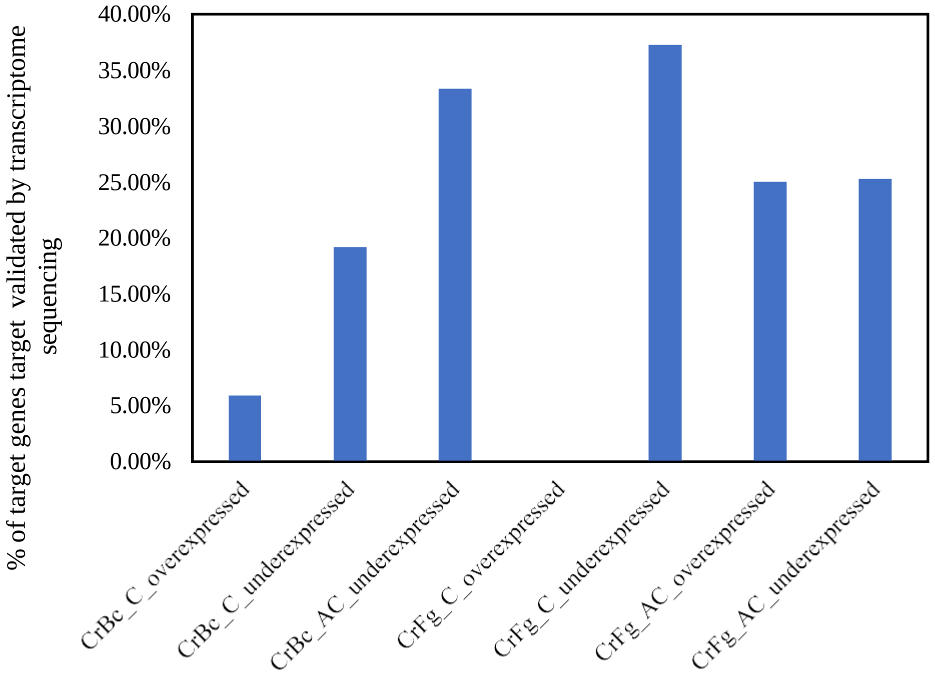

Supplementary figure S4: Validation degradome based gene targets by transcriptome sequencing. Percentage of genes, putatively cleaved by differentially expressed sRNAs in this study, which were also overexpressed in the Δ*dcl2* mutant during interaction with *B. cinerea* and *F. graminearum* in Piombo *et al.*, (2021).

Reference:

Piombo E, Vetukuri RR, Broberg A, Kalyandurg PB, Kushwaha S, Funck Jensen D, Karlsson M, Dubey M. 2021. Role of Dicer-dependent RNA interference in regulating mycoparasitic interactions. Microbiol Spectr 9:e01099-21.
